# Di-Isatropolone C, a Spontaneous Isatropolone C Dimer Derivative with Autophagy Activity

**DOI:** 10.3390/molecules29071477

**Published:** 2024-03-26

**Authors:** Jie Fu, Xiaoyan Liu, Miaoqing Zhang, Jiachang Liu, Shufen Li, Bingya Jiang, Linzhuan Wu

**Affiliations:** CAMS Key Laboratory of Synthetic Biology for Drug Innovation, NHC Key Laboratory of Biotechnology for Microbial Drugs, Institute of Medicinal Biotechnology, Chinese Academy of Medical Sciences & Peking Union Medical College, Beijing 100050, China; cfujie@126.com (J.F.); lxyzjk445@163.com (X.L.); zhangmiaoqing@imb.pumc.edu.cn (M.Z.); ljchang_1019@163.com (J.L.); lisf0229@163.com (S.L.)

**Keywords:** di-isatropolone C, isatropolone C, spontaneous dimerization, autophagy activity

## Abstract

Isatropolone C from *Streptomyces* sp. CPCC 204095 features a fused cyclopentadienone-tropolone-oxacyclohexadiene tricyclic moiety in its structure. Herein, we report an isatropolone C dimer derivative, di-isatropolone C, formed spontaneously from isatropolone C in methanol. Notably, the structure of di-isatropolone C resolved by NMR reveals a newly formed cyclopentane ring to associate the two isatropolone C monomers. The configurations of four chiral carbons, including a ketal one, in the cyclopentane ring are assigned using quantum NMR calculations and DP4+ probability. The plausible molecular mechanism for di-isatropolone C formation is proposed, in which complex dehydrogenative C-C bond coupling may have happened to connect the two isatropolone C monomers. Like isatropolone C, di-isatropolone C shows the biological activity of inducing autophagy in HepG2 cells.

## 1. Introduction

Isatropolones are a group of secondary metabolites from *Streptomyces*, with isatropolone C as a major component. Isatropolones share a fused cyclopentadienone-tropolone-oxacyclohexadiene tricyclic moiety and a deoxysugar moiety in their structures. Notably, they are able to conjugate non-enzymatically with amines or amino acids to generate isarubolones which share a fused cyclopentadienone-tropolone-pyridine tricyclic moiety [1,2].

Isatropolones/isarubrolones and their structurally similar natural products rubrolones and rubterolones are bioactive actinomycetes secondary metabolites [3,4,5,6]. They display biological activities against *Leishmania donovani*, inducing autophagy, protecting neonatal rat cardiomyocytes exposed to H_2_O_2_-induced injuries, and controlling potato common scab [1,3,4,5]. Thus, istropolones/isarubrolones, rubrolones, and rubterolones may have the potential to develop into drug-lead compounds, arousing interest in their structure diversification and biosynthesis.

So far, in vitro structure diversification has generated nearly one hundred derivatives/analogues of these metabolites with fused cyclopentadienone-tropolone-pyridine tricyclic moiety [6,7,8], and biosynthetic studies have demonstrated similar pathways and homologous gene clusters for isatropolones/isarubrolones, rubrolones, and rubterolones [1,8,9]. In particular, the tropolone ring in these metabolites is constructed via the oxygenase(s)-catalyzed rearrangement of poly-β-ketoacyl intermediates from type-II polyketide synthase (PKS) pathways.

Dimerization is a common strategy in the structure diversification and complexification of natural products, and many microbial secondary metabolites, such as actinorhodin, echinomycin, and dibohemamine, are dimers in which two monomers are often associated via C–C, C–O, or C–N bonds [10,11,12].

**Figure 1:**
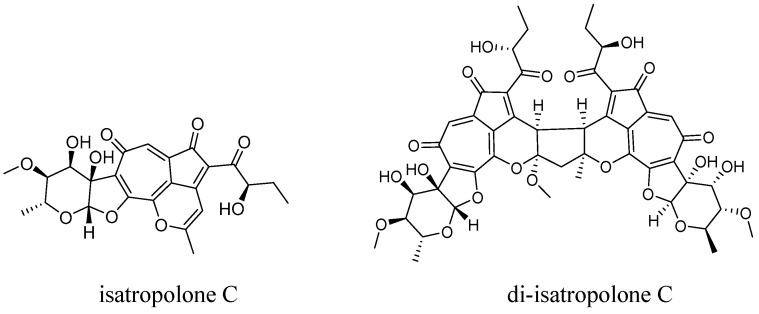
Chemical structure of isatropolone C and di-isatropolone C (**1**).

We are interested in novel secondary metabolites from actinomycetes. Previously, we identified 7,12-dihydroisatropolone C from the isatropolones/isarubrolones producer *Streptomyces* sp. CPCC 204095, proving it to be the immediate chemical precursor of isatropolone C [13]. Recently, we found that isatropolone C (Figure 1) was able to dimerize slowly and spontaneously in methanol, yielding novel isatropolone C dimer derivative(s) with a newly formed cyclopentane ring to associate the two isatropolone C monomers. The structure elucidation, formation, and autophagic activity of the isatropolone C dimer derivative produced from isatropolone C in methanol are mainly as described below.

## 2. Results and Discussion

### 2.1. Elucidation of Di-Isatropolone C Structure

A new peak was observed in the analytical HPLC of isatropolone C following incubation in methanol for ten or more days. The peak revealed a similar but different UV–visible absorption profile compared with isatropolone C (Figure S1). Furthermore, the peak revealed a molecular mass of about two times that of isatropolone C. Compound (**1**) in the peak aroused our interest, and thus it was purified for NMR structure elucidation.

Compound **1** was obtained as a yellow amorphous powder. Its molecular formula was determined as C_49_H_50_O_21_ by HRESIMS (*m*/*z* 975.2911 [M + H]^+^, calcd. for 975.2917, Figure S2). A molecular formula comparison suggested that **1** (with 25 degrees of unsaturation) may be an isatropolone C (C_24_H_24_O_10_, with 13 degrees of unsaturation) dimer plus CH_2_O. A UV–visible absorption profile comparison suggested that **1** should possess conjugated system(s) shorter than that of isatropolone C.

The ^1^H NMR spectral data of **1** (Figure S3) exhibited proton signals corresponding to five methyls [δ_H_ 0.81 t (7.2), 0.80 t (7.2), 1.27 d (6.0), 1.27 d (6.6), and 1.49 s] and three methoxy groups [δ_H_ 3.43, 3.36, 3.36; each 3H, s)]. A comprehensive analysis of the ^1^H, ^13^C, and HSQC NMR data of **1** (Figures S4–S7) revealed the presence of 49 carbons, including 22 pairs of signals. Among these paired signals, three pairs were ketone carbons, eight pairs were olefinic carbons, three pairs were methyls, one pair was sp^3^ methylenes, six pairs were sp^3^ methines, and one pair was sp^3^ quaternary carbons. These paired signals constituted nearly two sets of carbon signals for isatropolone C, which led us to conclude that **1** should be an isatropolone C dimer derivative. Correlations of 2D NMR spectra confirmed the presence of two isatropolone C monomers in **1**, and the (two) deoxysugar moiety, the (two) tropolone ring, and the (two) cyclopentadienone ring in **1** were identical to those in isatropolone C.

**Figure 2:**
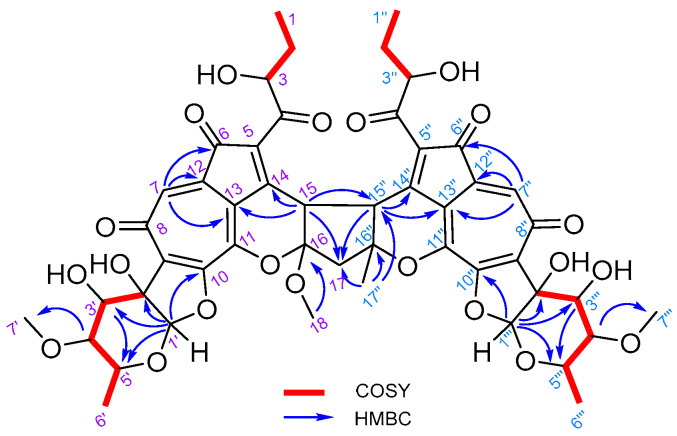
Key HMBC and COSY correlations of di-isatropolone C (**1**).

Five new sp^3^ carbons (δ_C_ 47.2, 48.3, 53.6, 89.2, and 112.5) emerged in **1**, while the sp^2^ carbons C-15 and C-16 (from the trisubstituted-double bond of pyran ring) in isatropolone C were lost. Four of the five new sp^3^ carbons were supposed to come from C-15 and C-16 in (two) isatropolone C, which were supported by HMBC correlations of H-15 to C-13 and C-14, and H-15″ to C-13″ and C-14″. The HMBC correlation of H_3_-18 to C-16 indicated that a methoxy group (the fifth new sp^3^ carbon, δ_C_ 53.6) was attached to C-16 of **1** (Figure 2).

The HMBC correlations of H-15 to C-15″ and C-17, H-15″ to C-15 and C-17″, H-17 to C-16, C-16″ and C-17″, and H-17″ to C-17, C-15″, and C-16″ suggested that C-17 as the methylene (-CH_2_-) bridge closed and thus yielded a cyclopentane ring (C15, C16, C17, C15″, and C16″) to associate the two isatropolone C monomers in **1**. Hence, the structure of **1** was resolved as 16-methoxy isatropolone C dimer derivative (Figure 1). For simplicity, **1** was designated as di-isatropolone C. The NMR data of di-isatropolone C were assigned completely in Table 1. As expected, di-isatropolone C possesses two conjugated systems, and each one is one C-C double bond shorter than that of isatropolone C. It is also noteworthy that there is a ketal carbon (C16) in the newly formed cyclopentane ring of di-isatropolone C.

Di-isatropolone C should be a stereo-specific isatropolone C dimer derivative due to its single set of NMR signals for the four new chiral carbons, C-15, C-16, C-15″, and C-16″, in the cyclopentane ring. However, the configurations of these chiral carbons were still not assigned. Efforts were unsuccessfully put into the crystallization of di-isatropolone C to elucidate its stereo-structure via the use of X-ray diffraction or MicroED. Therefore, quantum NMR calculations and DP4+ probability analyses were conducted to determine the configurations of C-15, C-16, C-15″, and C-16″ (Figures S9 and S10, Tables S1–S18). Among the 16 probable diastereomers of di-isatropolone C (from **a** to **p**), the **p** diastereomer revealed a DP4+ probability of 99.86% (Table S17). In particular, all (four) side atoms or groups, i.e., hydrogen at C15, methoxy at C16, hydrogen at C-15″, and methyl at C-16″, were out-positioned in the conformers of **p** diastereomer, which should favor the close-stacking of the two cyclopentadienone-tropolone planar moieties in di-isatropolone C (Table S18, Figure S11). Furthermore, H-15 and H-15″ were supported at the same side by the NOESY correlation between H-15(δ 4.23) and H-15″(δ 4.45) and by the inferred coupling constant 12.6 Hz between H-15 and H-15″ (Figure S8). As a stereo-specific isatropolone C dimer derivative, di-isatropolone C revealed a specific rotation ${\left[\alpha \right]}_{D}^{20}$ + 64 (c 0.2, MeOH).

### 2.2. Exploration of the Mechanisms of Di-Isatropolone C Formation

It is very interesting that di-isatropolone C is a regio-specific isatropolone C dimer derivative. A simple structure comparison suggests that the formation of di-isatropolone C from isatropolone C in methanol must have involved chemical reactions (or processes) of C-C bond formation, ketalization, and oxidation/dehydrogenation. As isatropolone C can be easily hydrated and the resulting isatropolone C hydrate may provide both carbonyl groups for ketalization and (active) α-H for C-C bond formation, the hydration of isatropolone C must have occurred before the dimerization of isatropolone C. In addition, the 16-methoxy group that appeared in ketalization should come from methanol, as 16-ethoxy di-isatropolone C was produced from isatropolone C in ethanol, and then purified and identified using NMR (Figures S12–S19, Table S19).

We explored some factors affecting di-isatropone C formation, which may help us propose a plausible molecular mechanism of di-isatropolone C formation. First, when isatropolone C was dissolved in non-protic solvents, such as acetone or acetonitrile, di-isatropolone C or isatropolone C dimer derivative(s) was not observed (Figure S21), which suggested that protic solvents such as methanol were essential for di-isatropolone C formation. Second, when isatropolone C was dissolved in methanol supplemented with TEMPO (2,2,6,6-tetramethylpiperidoxyl), a radical scavenger and catalyst for the oxidation of alcohols to aldehydes or ketones [14], di-isatropolone C formation was significantly improved (Figure S22), which suggested that the oxidation/dehydrogenation of alcohol(s) to ketones may have happened in di-isatropolone C formation. Third, when isatropolone C was dissolved in methanol (and supplemented with TEMPO) and placed in an oxygen-free incubator, di-isatropolone C was not observed (Figure S23), proving that oxygen was essential for di-isatropolone C formation.

**Figure 3:**
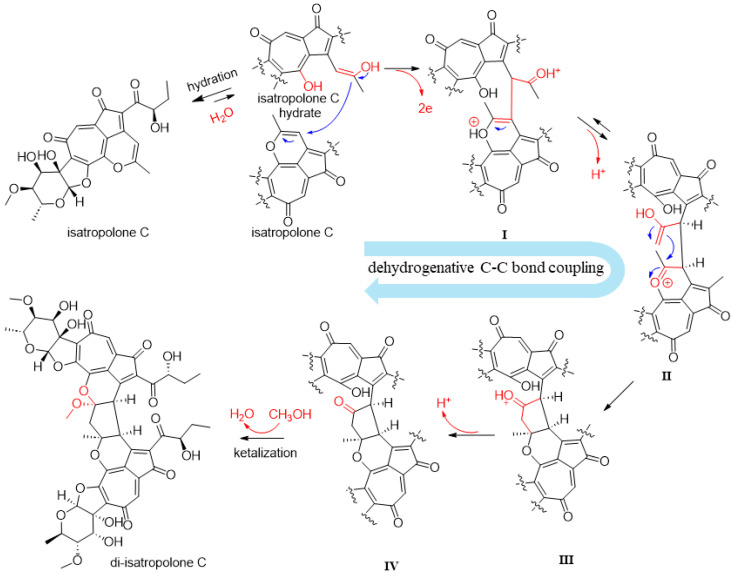
The plausible molecular mechanism of di-isatropolone C formation. First, one molecule of isatropolone C is hydrated, which yields the enolate form of isatropolone C hydrate. The isatropolone C hydrate approaches isatropolone C by intermolecular hydrophobic and π-π interactions of the planar cyclopentadienone-tropolone and cyclopentadienone-tropolone-oxacyclohexadiene moieties. Then, the isatropolone C hydrate undergoes complex dehydrogenative coupling with the close-stacked isatropolone C, which results in intermediate **IV** with two C-C bonds to connect two isatropolone C monomers. The two electrons and the two protons released from the complex dehydrogenative coupling are presumed to combine with oxygen [O] from O_2_ to produce water molecule(s). Finally, intermediate **IV** is transformed into di-isatropolone C via ketalization at C-15 with the C-11 hydroxyl group and methanol.

Based on the above structure comparison and experimental results, we proposed a plausible molecular mechanism for di-isatropolone C formation, in which complex dehydrogenative C-C bond coupling may have happened to connect the two isatropolone C monomers (Figure 3). The coupling, especially the formation of the first C-C bond together with the release of two electrons to generate putative intermediate **I**, may be accelerated by TEMPO. However, details of the coupling were not confirmed, as no putative intermediates (**I–IV**) from the coupling have been experimentally confirmed. We believe that computational chemistry studies may be conducted to help elucidate mechanism details in the dehydrogenative C-C bond coupling and thermodynamics of the spontaneous di-isatropolone C formation.

### 2.3. Autophagy Activity of Di-Isatropolone C

**Figure 4:**
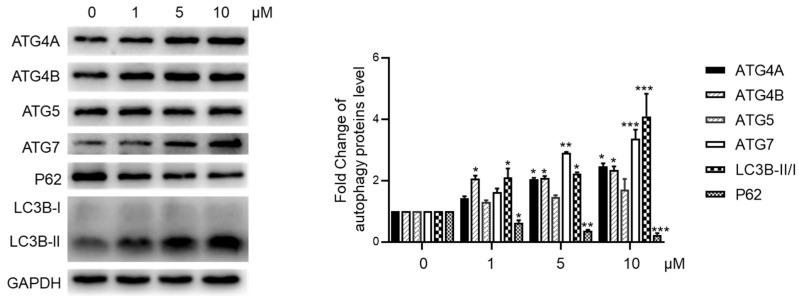
Di-isatropolone C activated autophagy in HepG2. The levels of autophagy proteins ATG4A/B, ATG5, ATG7, P62, and LC3B-II/I ratio in HepG2 cells were displayed using Western blotting. GADPH (glyceraldehyde-3-phosphate dehydrogenase) was used as a loading control. * *p* < 0.05, ** *p* < 0.01, and *** *p* < 0.001 vs. di-isatropolone C at 0 μM treatment group. The statistics data were expressed as mean ± standard deviation (n = 5).

We assessed the autophagy-inducing activity of di-isatropolone C, given the known autophagy activation properties of isatropolone C [1,3]. In a concentration-dependent manner, di-isatropolone C significantly increased the expression of ATG4B and ATG7, along with elevating the LC3B-II/I ratio, while notably reducing the expression of P62 in HepG2 cells (Figure 4). However, di-isatropolone C elevated ATG4A expression without significantly affecting ATG5. In contrast, isatropolone C downregulated ATG4A and upregulated ATG5 expression. These findings suggest that di-isatropolone C also possesses autophagy activation properties, albeit via a potentially distinct pathway compared with isatropolone C.

## 3. Materials and Methods

### 3.1. General Experimental Procedures

HPLC was conducted using the Agilent 1260 series with PDA detector (Agilent, Santa Clara, USA). For analytical HPLC, a reverse-phase C18 column (CAPCELL PAK C_18_ AQ from Shiseido, Tokyo, Japan; 250 mm × 4.6 mm, 5 μm) was used with a gradient solvent system from 15% to 70% ACN-H_2_O (0.1% HAc, *v*/*v*), 1.0 mL/min. For semi-preparative HPLC, a reverse-phase C_18_ column (CAPCELL PAK Pheny: 250 mm × 10 mm, 5 μm) was used with an isocratic solvent system of 37% ACN-H_2_O (0.1% HAc, *v*/*v*), 1.5 mL/min. HRESIMS data were obtained from Waters Xevo G2-XS QTof. NMR data were collected using a Bruker Avance III HD 600 MHz spectrometer (Bruker, Rheinstetten, Germany) with 16 scans, with pulse sequence zg30, at room temperature, using CD_3_OD as solvent, using TMS as an internal standard, and a sample concentration of ca. 12 mg/mL. In particular, pulse sequences for 2D NMR including COSY, HSQC, HMBC, and NOESY were cosygpmfqf, hsqcedetgpsisp2.3, hmbcgpndqf, and noesygpphpp, respectively. The optical rotation was conducted using a JASCO P-2000 spectrometer (JASCO, Easton, MD, USA).

### 3.2. Preparation of Isatropolone C

Frozen stock spores of *Streptomyces* sp. CPCC 204095 were thawed, inoculated on the culture medium (soluble starch 1.0%, yeast extract 0.4%, malt extract 1.0%, glucose 0.4%, and agar 1.5%), and incubated at 28 °C for 7 days for sporulation. Fresh spores were collected and spread on the fermentation medium (yeast extract 0.4%, malt extract 2.5%, glucose 0.4%, soybean cake 0.6%, and agar 1.5%) plates and incubated at 28 °C for 30–36 h for isatropolone C production. The agar culture (5 L) was then collected and extracted with EtOAc twice. The combined organic layer was vacuum dried, yielding a dark brown residue (6.6 g). The residue was loaded onto a preparative silica column for fractionation with CH_2_Cl_2_-MeOH (3% CH_2_Cl_2_-MeOH, 30 min; 4% CH_2_Cl_2_-MeOH, 15 min; 6% CH_2_Cl_2_-MeOH, 60 min; 8% CH_2_Cl_2_-MeOH, 10 min; 15% CH_2_Cl_2_-MeOH, 15 min; *v*/*v*) at a constant flow rate of 35 mL/min, which yielded five fractions from F1-1 to F1-5. Each fraction was analyzed using HPLC. Fractions containing isatropolone C were combined and concentrated under reduced pressure, yielding a dark brown residue. The residue was loaded onto a preparative ODS column for fractionation with MeOH-H_2_O (25% MeOH-H_2_O, 30 min; 30% MeOH-H_2_O, 20 min; 35% MeOH-H_2_O, 126 min; H_2_O contained 0.1% HAc, *v*/*v*) at a constant flow rate of 25 mL/min, which yielded four fractions from F2-1 to F2-4. Each fraction was analyzed using HPLC. Fractions F2-2 and F2-3 were found to contain isatroplone C. They were combined and vacuum-dried, which resulted in ca. 650 mg of isatropolone C with purity levels ≥ 90% using analytical HPLC [15].

### 3.3. Production and Purification of Di-Isatropolone C

Isatropolone C was dissolved in methanol and incubated at room temperature (20–25 °C) for 15 days. The analytical HPLC of the isatropolone C solution revealed a new peak at 17.5 min (13.9 min for isatropolone C). The compound in the new peak was purified using semi-preparative HPLC. A pure preparation of 2.8 mg compound **1** (di-isatropolone C) was obtained from 150 mg isatropolone C in 10.0 mL methanol.

### 3.4. Quantum NMR Calculations and DP4+ Probability Analysis

There are a total of four unassigned chiral carbons (C-15, C-16, C-15″, and C-16″) in di-isatropolone C, which results in a total number of sixteen (2^4^) probable diastereomers for di-isatropolone C. Quantum NMR calculations were employed to assign the absolute configurations of the four chiral carbons in di-isatropolone C. Conformational analysis was performed using OpenBabel 2.4.1 with genetic algorithms at the MMFF94 force field for the sixteen diastereomers of di-isatropolone C [16]. The conformers of each diastereomer were then optimized with the software package Gaussian 09 at the M062X/6-31G(d) level [17]. Room-temperature equilibrium populations were calculated according to the Boltzmann distribution law. The energies and populations of the dominative conformers of the sixteen diastereomers were provided. The ^13^C NMR calculations were carried out using Gaussian 09, following the protocol adapted from Lodewyk et al. [18]. In detail, the theoretical calculations of ^13^C NMR were conducted using the Gauge-Including Atomic Orbitals (GIAO) method at mPW1PW91/6-31G(d) in chloroform through the use of the SMD Solvation model. To remove systematic errors resulting from the conformational search and random errors from experimental conditions, the calculated ^13^C NMR chemical shifts were averaged according to the Boltzmann distribution for each conformer and fitted to the experimental data via ordinary least squares linear regression analysis. Finally, DP4+ probability analysis was performed as described by Grimblat et al. [19].

### 3.5. Factors Affecting Di-Isatropone C Production

Solvent: Isatropolone C was dissolved in 400 μL methanol, acetonitrile, or acetone at a concentration of 0.8, 0.8, and 0.4 mg/mL, respectively. The solutions were incubated at 4 °C for 30 days, and then analyzed using HPLC for di-isatropolone C production.

TEMPO: Isatropolone C was dissolved in methanol at a concentration of 0.8 mg/mL, and TEMPO was dissolved in H_2_O at a concentration of 10.0 mg/mL. The isatropolone C solution (200 μL) was added with 1.0 μL TEMPO solution. The mixed solution was incubated at 4 °C for 30 days, and then analyzed using HPLC for di-isatropolone C production. Meanwhile, an identical volume of isatropolone C solution without TEMPO was incubated at 4 °C for 30 days as the control.

Oxygen: Isatropolone C was dissolved in degassed methanol at a concentration of 0.8 mg/mL, and TEMPO was dissolved in degassed H_2_O at a concentration of 10.0 mg/mL. The isatropolone C solution (400 μL) was added with 5.0 μL TEMPO solution. The mixed solution was incubated anaerobically (under a gas mixture of nitrogen 90%, carbon dioxide 5%, and hydrogen 5%) at 37 °C for 30 days, and then analyzed using HPLC for di-isatropolone C production. Meanwhile, an identical volume of the mixed solution was incubated aerobically (under air) at 37 °C for 30 days as control.

### 3.6. Autophagy Activity Assay

HepG2 cells were cultured in Minimum Essential Media (MEM) (Gibco) containing 10% fetal bovine serum (FBS) (Gibco) with penicillin (100 μg/mL) and streptomycin (100 μg/mL). They were treated with isatropolone C for 24 h at final concentrations of 0, 1, 5, or 10 μM. The cells were then lysed in a RIPA cell lysis buffer with a protease inhibitor cocktail. Proteins were separated using 12% SDS-PAGE and transferred onto nitrocellulose membranes for the quantification of autophagy proteins P62, LC3B-II/I, ATG4a/b, ATG7, and ATG5 via the use of Western blotting. The membranes were incubated with anti-LC3B (M186-3, MBL), anti-P62 (PM045, MBL), anti-ATG5 (NB110-53818, Novus), anti-ATG7 (8558S, Cell Signaling Technology), anti-ATG4a (7613S, Cell Signaling Technology), and anti-ATG4b antibodies (13507S, Cell Signaling Technology) for the quantification of autophagy marker proteins ATG4a/b, ATG5, ATG7, LC3B, and P62. GADPH (glyceraldehyde-3-phosphate dehydrogenase) was used as a loading control. Data analysis was performed using GraphPad Prism 8.0 software. Statistical comparisons were conducted using one-way analysis of variance (ANOVA) and Student’s *t*-test as appropriate. The data were shown as mean ± standard deviation based on data obtained from five independent experiments. A significance level of *p* < 0.05 was considered statistically significant.

## Figures and Tables

**Table 1:** NMR data of di-isatropolone C in methanol-*d*_4_.

Position	*δ*_C_, Type	*δ*_H_, Mult, (*J* in Hz)	Position	*δ*_C_, Type	*δ*_H_, Mult, (*J* in Hz)
1	9.9, CH_3_	0.81 t (7.2)	1″	9.9, CH_3_	0.80 t (7.2)
2	27.4, CH_2_	1.23 m, 1.55 m	2″	27.4, CH_2_	1.23 m, 1.59 m
3	77.3, CH	4.69 dd (7.8, 3.6)	3″	77.4, CH	4.72 dd (7.8, 3.6)
4	199.3, C		4″	199.5, C	
5	130.2, C		5″	130.8, C	
6	191.3, C		6″	191.5, C	
7	130.6, CH	6.99 s	7″	131.5, C	7.02 s
8	185.5, C		8″	185.5, C	
9	132.5, C		9″	132.7, C	
10	159.0, C		10″	159.1, C	
11	145.5, C		11″	147.3, C	
12	134.2, C		12″	134.6, C	
13	120.4, C		13″	122.2, C	
14	167.0, C		14″	167.4, C	
15	47.2, CH	4.23 d (12.6)	15″	48.3, CH	4.45 d (13.2)
16	112.5, C		16″	89.2, C	
17	48.2, CH_2_	2.88 d (16.2) 3.04 d (15.6)	17″	22.5, CH_3_	1.49 s
18	53.6, CH_3_	3.43 s			
1′	109.9, CH	5.64 d (4.8)	1‴	110.0, CH	5.64 d (4.8)
2′	82.7, C		2‴	82.7, C	
3′	69.1, CH	4.30 d (3.0)	3‴	69.1, CH	4.32 d (3.0)
4′	81.0, CH	3.28 m overlap	4‴	81.0, CH	3.28 m overlap
5′	68.0, CH	4.03 m	5‴	68.3, CH	4.03 m
6′	18.5, CH_3_	1.27 d (6.0)	6‴	18.6, CH_3_	1.27 d (6.6)
7′	57.7, CH_3_	3.36 s	7‴	57.8, CH_3_	3.36 s

## Data Availability

Data are contained within the article and Supplementary Materials.

## Supplementary Materials

The following supporting information can be downloaded at https://www.mdpi.com/article/10.3390/molecules29071477/s1, Figure S1. HPLC of isatropolone C and di-isatropolone C (**1**) with their UV-visible spectra. Figure S2. HRESIMS of di-isatropolone C (**1**). Figures S3–S8. NMR spectra of di-isatropolone C (**1**). Figure S9. Chemical structures of all 16 probable diastereomers of di-isatropolone C. Tables S1–S16. Energies and populations of conformers of diastereomer **a**–**p**. Figure S10. Linear regression fitting of computed ^13^C NMR chemical shifts of 16 diastereomers of di-isatropolone C (**1**) with experimental data. Table S17. The results of experimental and computed ^13^C NMR chemical shifts comparison and DP4+ probability analysis of di-isatropolone C (**1**). Table S18. Conformers of diastereomer **p**. Figure S11. The three most populated conformers of diastereomer **p**. Figure S12. The structure of 16-ethoxy di-isatropolone C. Figure S13. HPLC of isatropolone C and 16-ethoxy di-isatropolone C with their UV-visible spectra. Figure S14. HRESIMS of 16-ethoxy di-isatropolone C. Figures S15–S19. NMR spectra of 16-ethoxy di-isatropolone C. Table S19. NMR data of 16-ethoxy di-isatropolone C in methanol-*d*_4_. Figure S20. A time-course monitoring of di-isatropolone C production from isatropolone C in methanol incubated at 4 °C or room temperature. Figure S21. Production of di-isatropolone C from isatropolone C in methanol, acetonitrile, or acetone. Figure S22. Production of di-isatropolone C from isatropolone C in methanol with TEMPO. Figure S23. Production of di-isatropolone C from isatropolone C in methanol under air (aerobic) or oxygen-free gas mixture (anaerobic).
