# Supplementary material for: Di-Isatropolone C, a Spontaneous Isatropolone C Dimer Derivative with Autophagy Activity
Source: Molecules. 2024 Mar 26;29(7):1477. doi: 10.3390/molecules29071477 (PMC11013608; doi:10.3390/molecules29071477)
Supplement: Supplementary file 1 [file molecules-29-01477-s001.zip › molecules-2916206-supplementary.pdf]

# Supplementary Materials for

## Di-isatropolone C, a spontaneous isatropolone C dimer derivative with autophagy activity

Jie Fu, Xiaoyan Liu, Miaoqing Zhang, Jiachang Liu, Shufen Li, Bingya Jiang*, Linzhuan Wu*

CAMS Key Laboratory of Synthetic Biology for Drug Innovation, NHC Key Laboratory of Biotechnology for Microbial Drugs, Institute of Medicinal Biotechnology, Chinese Academy of Medical Sciences & Peking Union Medical College, Beijing 100050, China

* Bingya Jiang - Institute of Medicinal Biotechnology, Chinese Academy of Medical Sciences and Peking Union Medical College, Beijing 100050, P. R. China; Email: jiangbingya@163.com

* Linzhuan Wu - Institute of Medicinal Biotechnology, Chinese Academy of Medical Sciences and Peking Union Medical College, Beijing 100050, P. R. China; Email: wulinzhuan@imb.pumc.edu.cn

Jie Fu and Xiaoyan Liu contributed equally to the paper.

# Contents

**Part 1. Structure elucidation of di-isatropolone C (1)**

**Figure S1.** HPLC of isatropolone C and di-isatropolone C (**1**) with their UV-visible spectra ........ 3

**Figure S2.** HRESIMS of di-isatropolone C (**1**) ........ 4

**Figure S3.** $^{1}$H NMR spectrum of di-isatropolone C (**1**) ........ 4

**Figure S4.** $^{13}$C NMR spectrum of di-isatropolone C (**1**) ........ 5

**Figure S5.** $^{1}$H-$^{1}$H COSY spectrum of di-isatropolone C (**1**) ........ 5

**Figure S6.** HSQC spectrum of di-isatropolone C (**1**) ........ 6

**Figure S7.** HMBC spectrum of di-isatropolone C (**1**) ........ 7

**Figure S8.** NOESY spectrum of di-isatropolone C (**1**) ........ 7

**Part 2. Determination of configurations of chiral carbons C-15, C-16, C-15'' and C-16'' in di-isatropolone C (1)**

**Figure S9.** Chemical structures of all 16 probable diastereomers of di-isatropolone C .... 9

**Table S1.** Energies and populations of conformers of diastereomer **a** ........ 10

**Table S2.** Energies and populations of conformers of diastereomer **b** ........ 10

**Table S3.** Energies and populations of conformers of diastereomer **c** ........ 11

**Table S4.** Energies and populations of conformers of diastereomer **d** ........ 11

**Table S5.** Energies and populations of conformers of diastereomer **e** ........ 11

**Table S6.** Energies and populations of conformers of diastereomer **f** ........ 12

**Table S7.** Energies and populations of conformers of diastereomer **g** ........ 12

**Table S8.** Energies and populations of conformers of diastereomer **h** ........ 13

**Table S9.** Energies and populations of conformers of diastereomer **i** ........ 14

**Table S10.** Energies and populations of conformers of diastereomer **j** ........ 15

**Table S11.** Energies and populations of conformers of diastereomer **k** ........ 15

**Table S12.** Energies and populations of conformers of diastereomer **l** ........ 16

**Table S13.** Energies and populations of conformers of diastereomer **m** ........ 17

**Table S14.** Energies and populations of conformers of diastereomer **n** ........ 17

**Table S15.** Energies and populations of conformers of diastereomer **o** ........ 18

**Table S16.** Energies and populations of conformers of diastereomer **p** ........ 19

**Figure S10.** Linear regression fitting of computed $^{13}$C NMR chemical shifts of 16 diastereomers of di-isatropolone C (**1**) with experimental data ........ 22

**Table S17.** The results of experimental and computed $^{13}$C NMR chemical shifts comparison and DP4+ probability analysis of di-isatropolone C (**1**) ........ 23

**Table S18.** Conformers of diastereomer **p** ........ 24

**Figure S11.** The three most populated conformers of diastereomer **p** (from Table S17 and S18) ........ 29

**Part 3. Structure elucidation of 16-ethoxy di-isatropolone C**

**Figure S12.** The structure of 16-ethoxy di-isatropolone C ........ 30

**Figure S13.** HPLC of isatropolone C and 16-ethoxy di-isatropolone C with their UV-visible spectra ........ 30

**Figure S14.** HRESIMS of 16-ethoxy di-isatropolone C ........ 31

**Figure S15.** $^{1}$H NMR spectrum of 16-ethoxy di-isatropolone C ........ 32

**Figure S16.** $^{13}$C NMR spectrum of 16-ethoxy di-isatropolone C....................................32
**Figure S17.** $^{1}$H-$^{1}$H COSY spectrum of 16-ethoxy di-isatropolone C..............................33
**Figure S18.** HSQC spectrum of 16-ethoxy di-isatropolone C..........................................33
**Figure S19.** HMBC spectrum of 16-ethoxy di-isatropolone C.........................................34
**Table S19.** NMR data of 16-ethoxy di-isatropolone C in methanol-$d_4$..........................34
**Part 4. Factors affecting di-isatropone C (1) production**
**Figure S20.** A time-course monitoring of di-isatropolone C production from isatropolone C in methanol incubated at 4 °C or room temperature................................36
**Figure S21.** Production of di-isatropolone C from isatropolone C in methanol, acetonitrile, or acetone ...........................................................................37
**Figure S22.** Production of di-isatropolone C from isatropolone C in methanol with TEMPO......................................................................................................38
**Figure S23.** Production of di-isatropolone C from isatropolone C in methanol under air (aerobic) or oxygen-free gas mixture (anaerobic).......................................39

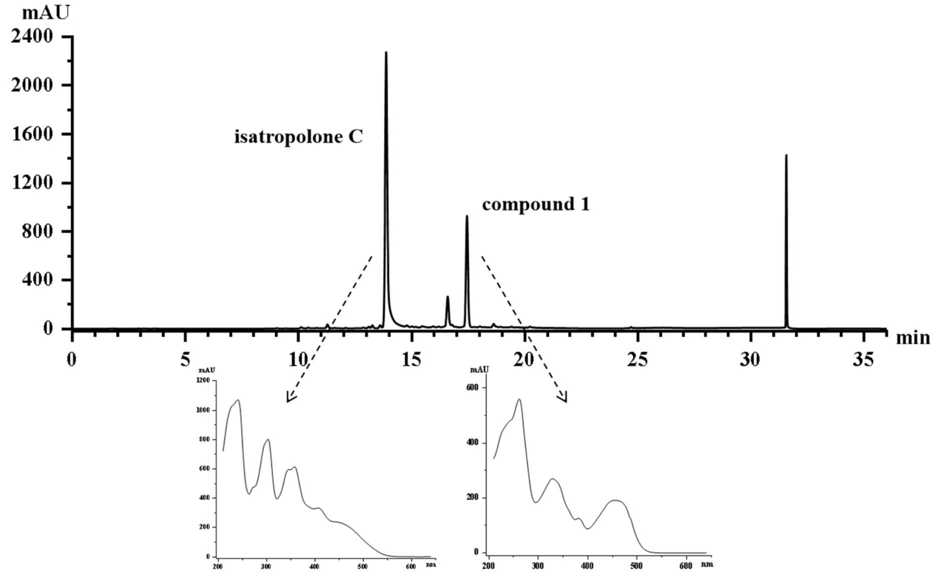


**Figure S1.** HPLC of isatropolone C and di-isatropolone C (**1**) with their UV-visible spectra

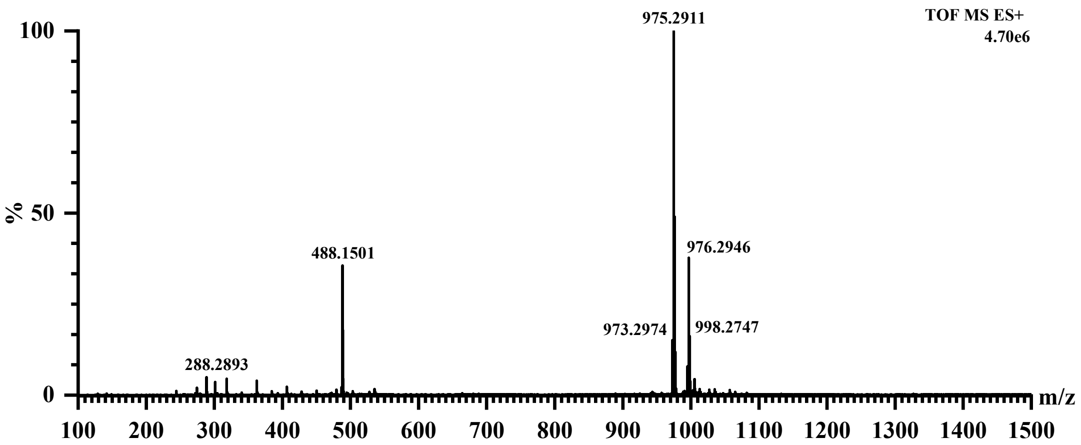


**Figure S2.** HRESIMS of di-isatropolone C (**1**)

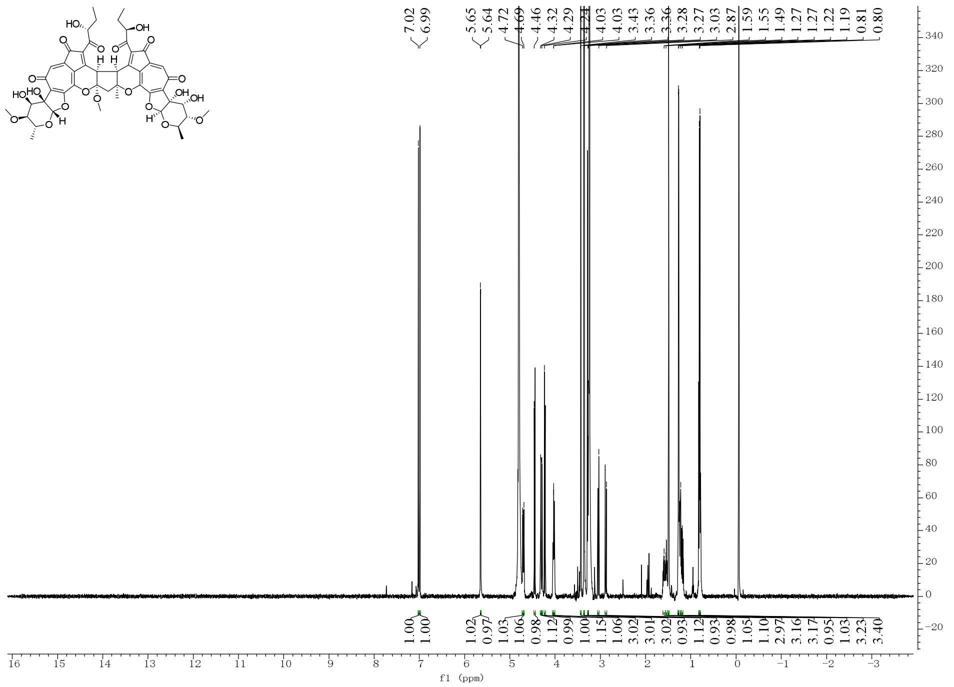


**Figure S3.** $^{1}$H NMR spectrum of di-isatropolone C (**1**)

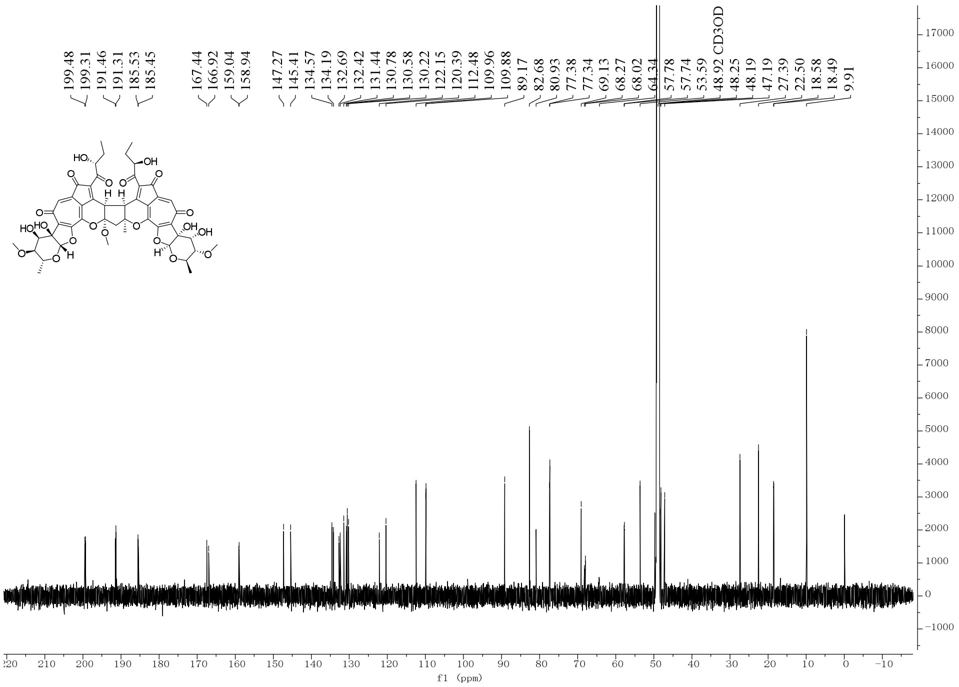


**Figure S4.** $^{13}$C NMR spectrum of di-isatropolone C (**1**)

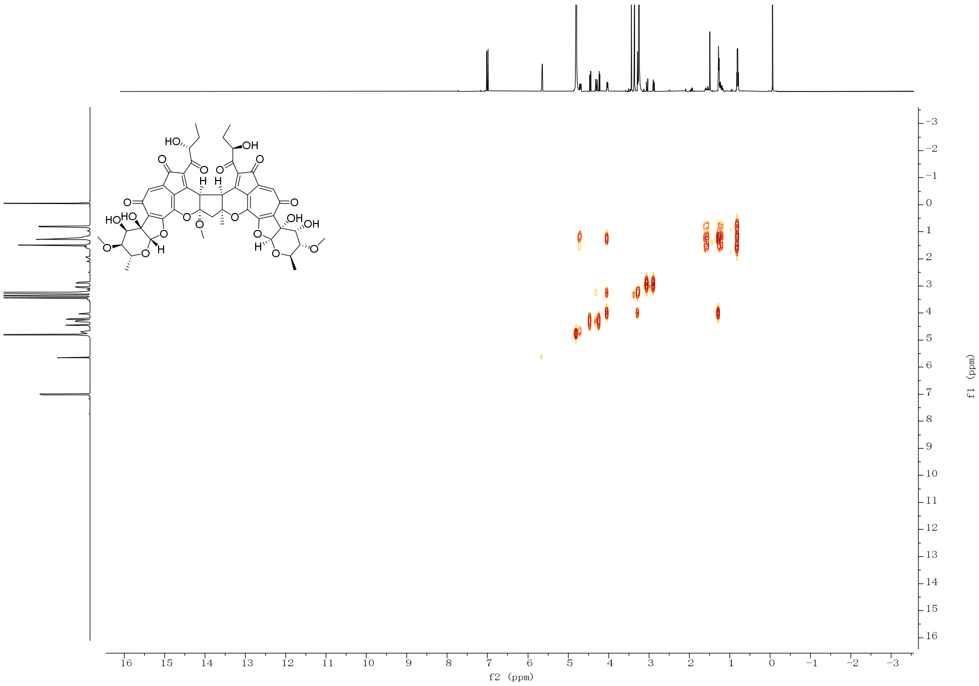


**Figure S5.** $^{1}$H-$^{1}$H COSY spectrum of di-isatropolone C (**1**)

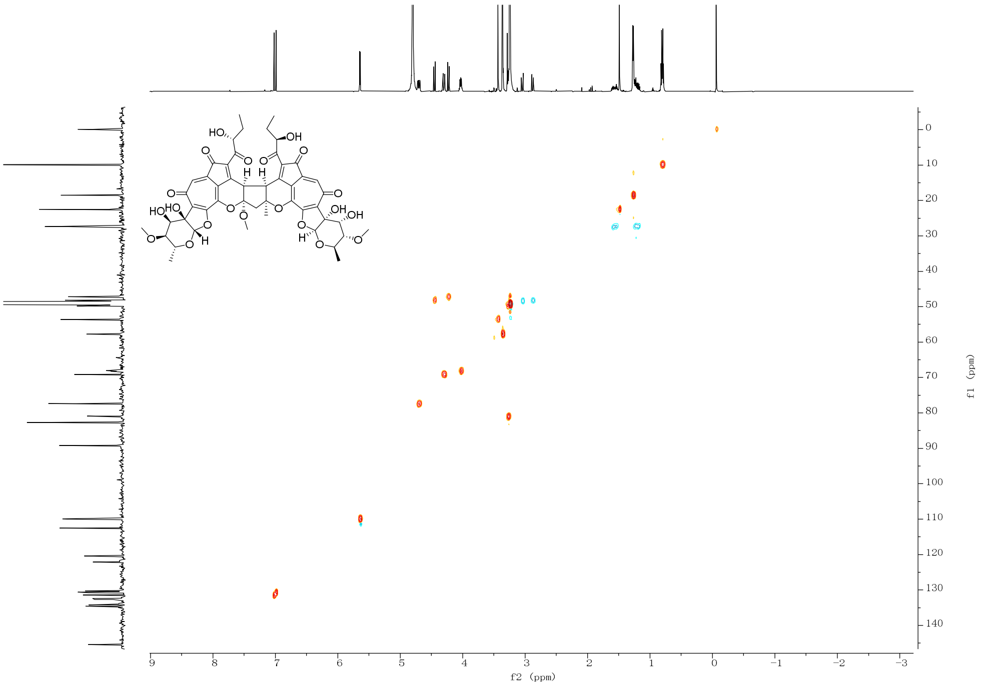


**Figure S6.** HSQC spectrum of di-isatropolone C (**1**)

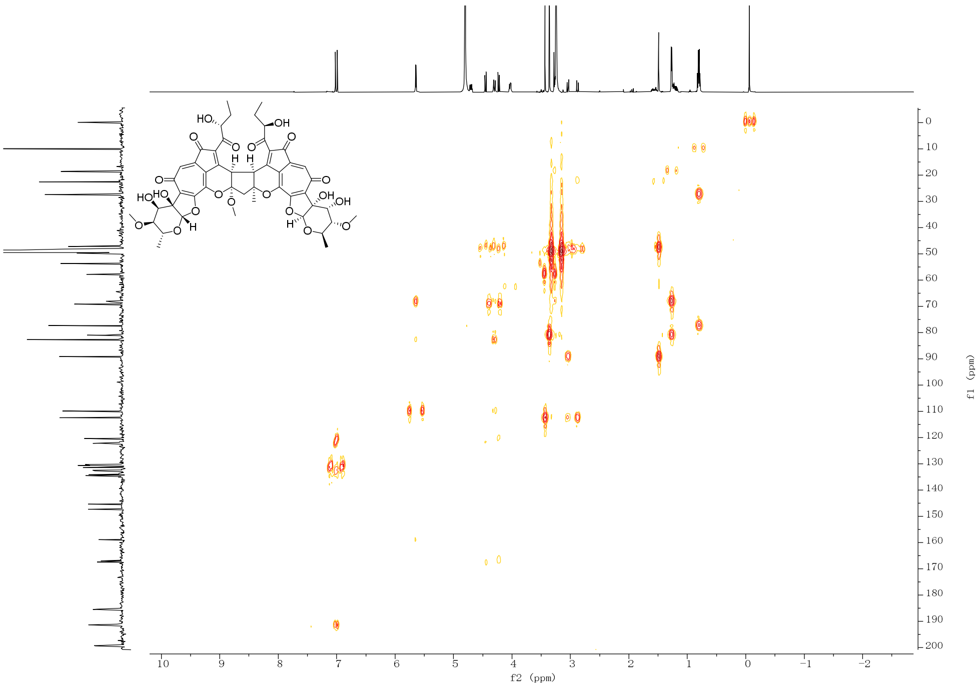


**Figure S7.** HMBC spectrum of di-isatropolone C (**1**)

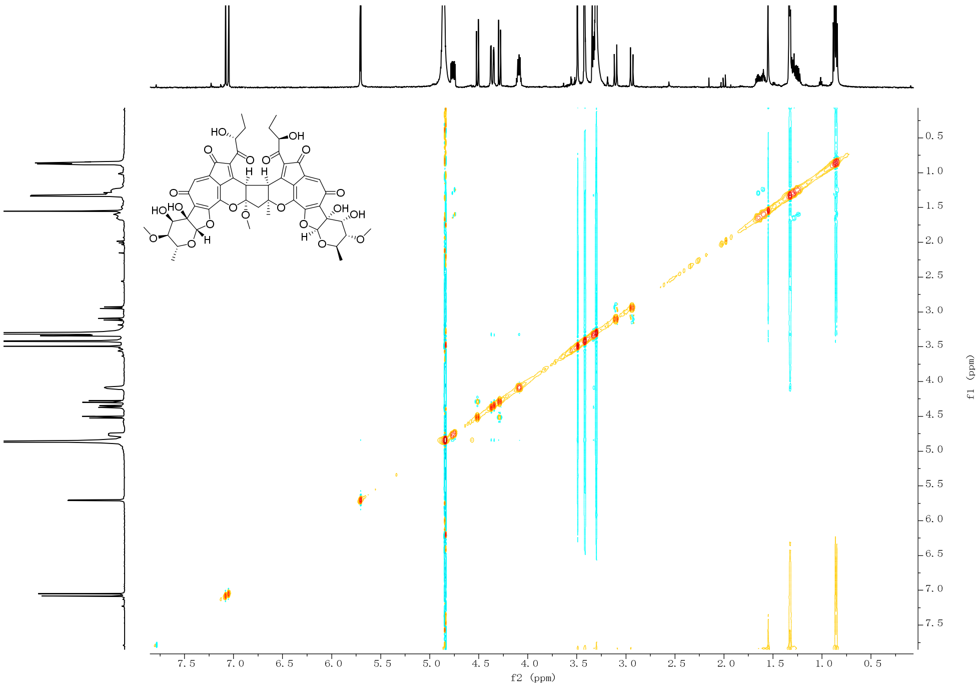


**Figure S8.** NOESY spectrum of di-isatropolone C (**1**)

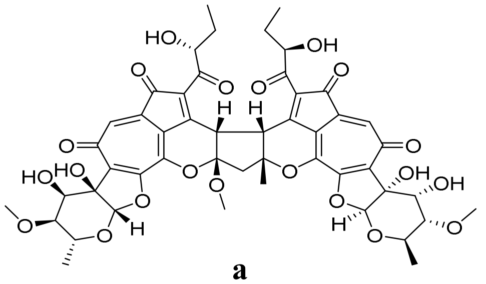

**a**

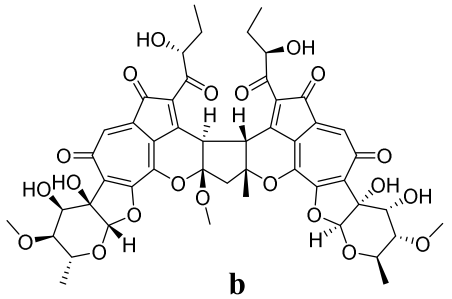

**b**

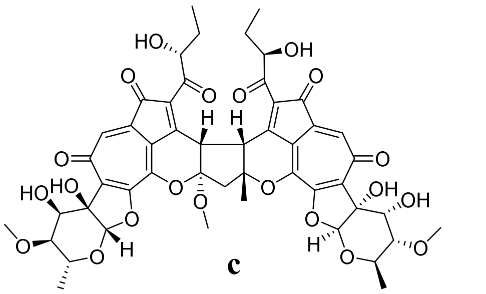

**c**

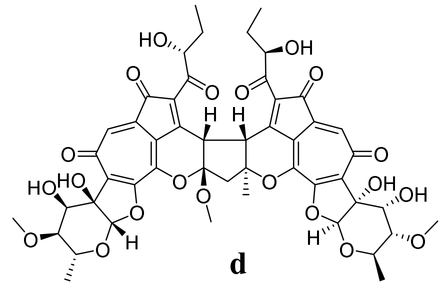

**d**

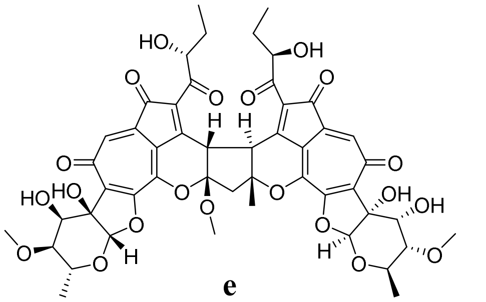

**e**

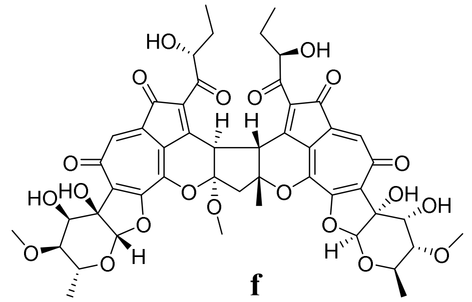

**f**

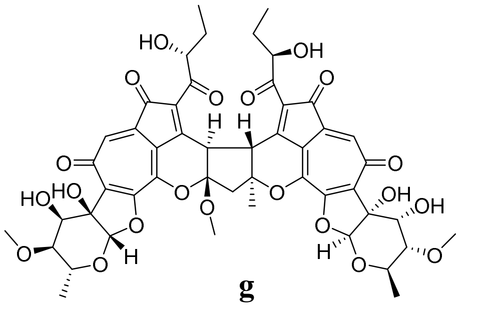

**g**

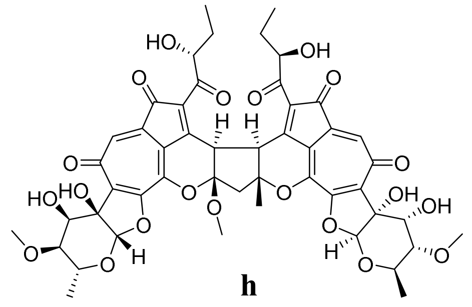

**h**

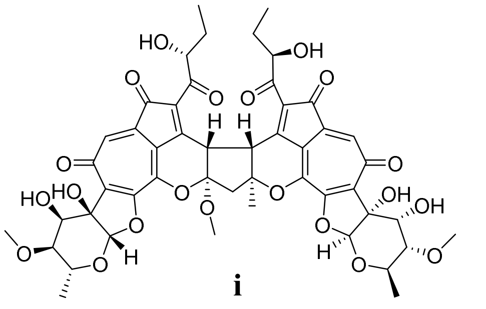

**i**

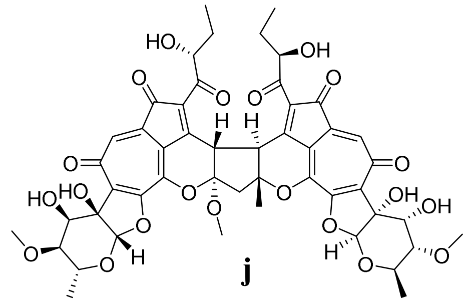

**j**

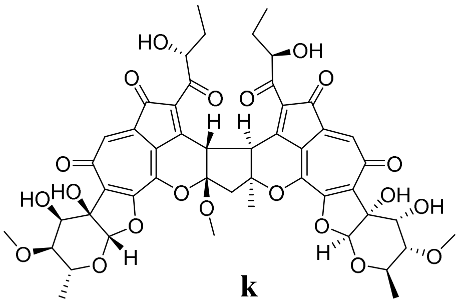

**k**

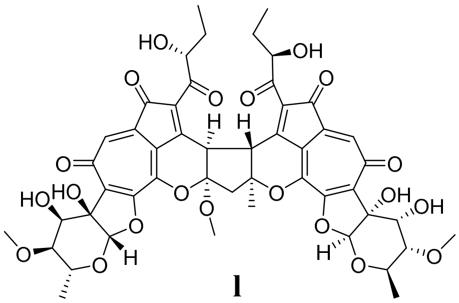

**l**

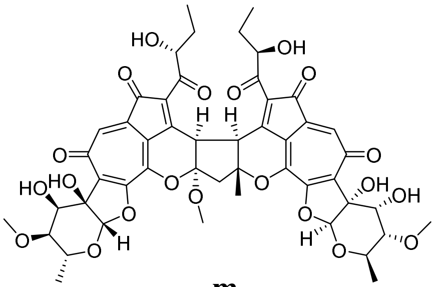

**m**

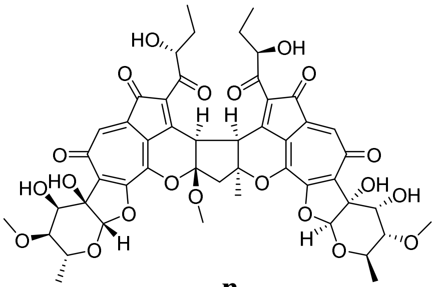

**n**

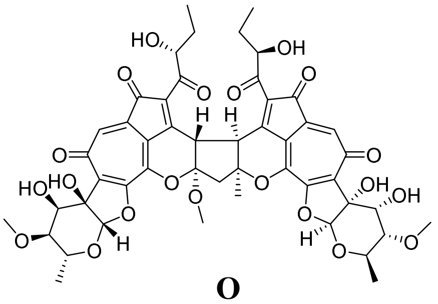

**O**

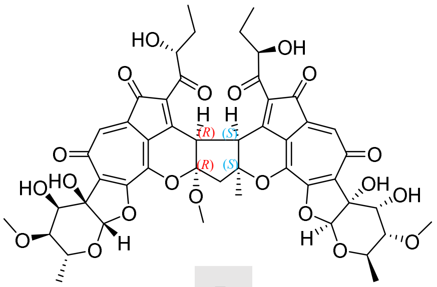

**P**

**Figure S9.** Chemical structures of all 16 probable diastereomers of di-isatropolone C

**Table S1.** Energies and populations of conformers of diastereomer **a**

| Conformer | Population (%) | E (Hartree) |
|---|---|---|
| 1 | 16.93 | -3475.590599 |
| 2 | 11.95 | -3475.590271 |
| 3 | 19.43 | -3475.590729 |
| 4 | 8.41 | -3475.589939 |
| 7 | 9.14 | -3475.590018 |
| 16 | 1.01 | -3475.587939 |
| 23 | 8.43 | -3475.589941 |
| 33 | 13.01 | -3475.590351 |
| 40 | 1.03 | -3475.58796 |
| 58 | 10.66 | -3475.590163 |

**Table S2.** Energies and populations of conformers of diastereomer **b**

| Conformer | Population (%) | E (Hartree) |
|---|---|---|
| 1 | 10.77 | -3475.553422 |
| 2 | 4.14 | -3475.552521 |
| 4 | 2.09 | -3475.551875 |
| 5 | 1.12 | -3475.551287 |
| 10 | 17.11 | -3475.553858 |
| 14 | 1.06 | -3475.551236 |
| 19 | 4.75 | -3475.552649 |
| 26 | 58.96 | -3475.555025 |

**Table S3.** Energies and populations of conformers of diastereomer **c**

| Conformer | Population (%) | E (Hartree) |
|---|---|---|
| 11 | 100 | -3476.846558 |

**Table S4.** Energies and populations of conformers of diastereomer **d**

| Conformer | Population (%) | E (Hartree) |
|---|---|---|
| 1 | 7.55 | -3475.549551 |
| 2 | 1.17 | -3475.547793 |
| 3 | 2.14 | -3475.54836 |
| 11 | 2.12 | -3475.548352 |
| 14 | 26.56 | -3475.550737 |
| 17 | 26.49 | -3475.550734 |
| 20 | 4.4 | -3475.54904 |
| 21 | 9.5 | -3475.549767 |
| 23 | 4.38 | -3475.549036 |
| 24 | 9.45 | -3475.549762 |
| 27 | 6.23 | -3475.549368 |

**Table S5.** Energies and populations of conformers of diastereomer **e**

| Conformer | Population (%) | E (Hartree) |
|---|---|---|
| 1 | 15.52 | -3475.555587 |
| 2 | 12.34 | -3475.555371 |
| 3 | 13.42 | -3475.55545 |
| 4 | 4.42 | -3475.554402 |
| 5 | 4.99 | -3475.554516 |

| | | |
|---|---|---|
| 6 | 3.9 | -3475.554283 |
| 7 | 1.42 | -3475.55333 |
| 8 | 3.86 | -3475.554273 |
| 11 | 6.63 | -3475.554785 |
| 12 | 4.4 | -3475.554397 |
| 15 | 4.49 | -3475.554418 |
| 16 | 4.31 | -3475.554378 |
| 19 | 8.6 | -3475.55503 |
| 21 | 2.92 | -3475.554012 |
| 22 | 1.93 | -3475.55362 |
| 24 | 5.57 | -3475.55462 |
| 25 | 1.29 | -3475.553242 |

**Table S6.** Energies and populations of conformers of diastereomer **f**

| Conformer | Population (%) | E (Hartree) |
|---|---|---|
| 1 | 1.01 | -3475.563137 |
| 11 | 1.56 | -3475.563545 |
| 12 | 65.56 | -3475.567071 |
| 14 | 1.94 | -3475.563753 |
| 16 | 20.67 | -3475.565983 |
| 19 | 1.63 | -3475.563587 |
| 22 | 7.62 | -3475.565041 |

**Table S7.** Energies and populations of conformers of diastereomer **g**

| Conformer | Population (%) | E (Hartree) |
|---|---|---|
| 1 | 17.77 | -3475.554308 |

| | | |
|---|---|---|
| 2 | 4.34 | -3475.552979 |
| 3 | 6.76 | -3475.553396 |
| 4 | 17.83 | -3475.554311 |
| 5 | 11.24 | -3475.553876 |
| 7 | 6.32 | -3475.553333 |
| 8 | 12 | -3475.553938 |
| 9 | 7.49 | -3475.553493 |
| 10 | 3.94 | -3475.552888 |
| 16 | 4.22 | -3475.552952 |
| 17 | 2.6 | -3475.552493 |
| 19 | 2.31 | -3475.552385 |
| 20 | 1.62 | -3475.552051 |
| 24 | 1.54 | -3475.551999 |

**Table S8.** Energies and populations of conformers of diastereomer **h**

| Conformer | Population (%) | E (Hartree) |
|---|---|---|
| 1 | 14.81 | -3475.533528 |
| 2 | 17.09 | -3475.533662 |
| 3 | 10.25 | -3475.53318 |
| 4 | 9.77 | -3475.533135 |
| 5 | 5.87 | -3475.532655 |
| 6 | 5.75 | -3475.532634 |
| 7 | 4.49 | -3475.532401 |
| 8 | 6.69 | -3475.532778 |
| 9 | 3.94 | -3475.532279 |
| 11 | 3.82 | -3475.532249 |
| 12 | 3.01 | -3475.532024 |
| 13 | 3.22 | -3475.532087 |

| | | |
|---|---|---|
| 16 | 1.27 | -3475.531208 |
| 20 | 1.46 | -3475.531339 |
| 22 | 2.15 | -3475.531706 |
| 37 | 3.7 | -3475.532219 |
| 57 | 1.46 | -3475.531339 |
| 60 | 1.27 | -3475.531208 |

**Table S9.** Energies and populations of conformers of diastereomer **i**

| Conformer | Population (%) | E (Hartree) |
|---|---|---|
| 1 | 10.98 | -3475.527922 |
| 2 | 10.12 | -3475.527845 |
| 3 | 13.99 | -3475.52815 |
| 4 | 15.98 | -3475.528276 |
| 5 | 6.87 | -3475.527479 |
| 10 | 4.49 | -3475.527079 |
| 13 | 6.25 | -3475.52739 |
| 18 | 1.38 | -3475.525968 |
| 23 | 1.3 | -3475.525912 |
| 24 | 9.65 | -3475.5278 |
| 28 | 2.15 | -3475.526384 |
| 35 | 6.22 | -3475.527387 |
| 36 | 2.79 | -3475.526631 |
| 49 | 1.79 | -3475.52621 |
| 58 | 6.04 | -3475.527358 |

**Table S10.** Energies and populations of conformers of diastereomer **j**

| Conformer | Population (%) | E (Hartree) |
|---|---|---|
| 1 | 30.26 | -3475.533573 |
| 2 | 17.97 | -3475.533081 |
| 3 | 20.26 | -3475.533194 |
| 5 | 11.53 | -3475.532662 |
| 6 | 5.48 | -3475.531961 |
| 7 | 6.07 | -3475.532056 |
| 8 | 3.51 | -3475.53154 |
| 12 | 1.14 | -3475.530479 |
| 13 | 1.45 | -3475.530706 |
| 14 | 1.17 | -3475.530501 |
| 17 | 1.16 | -3475.530499 |

**Table S11.** Energies and populations of conformers of diastereomer **k**

| Conformer | Population (%) | E (Hartree) |
|---|---|---|
| 2 | 11.55 | -3475.580585 |
| 5 | 4.42 | -3475.579679 |
| 6 | 3.76 | -3475.579527 |
| 8 | 3.03 | -3475.579325 |
| 10 | 1.83 | -3475.578847 |
| 13 | 1.83 | -3475.578849 |
| 14 | 1.42 | -3475.578607 |
| 21 | 4.69 | -3475.579735 |
| 24 | 11.02 | -3475.580541 |
| 26 | 1.28 | -3475.578509 |
| 29 | 1.42 | -3475.578606 |

| | | |
|---|---|---|
| 30 | 11.22 | -3475.580558 |
| 31 | 3.8 | -3475.579537 |
| 32 | 2 | -3475.578929 |
| 36 | 10.94 | -3475.580534 |
| 42 | 3.08 | -3475.579338 |
| 45 | 3.02 | -3475.579318 |
| 47 | 1.21 | -3475.578456 |
| 48 | 3.86 | -3475.579552 |
| 49 | 1.32 | -3475.578539 |
| 50 | 2.19 | -3475.579018 |
| 55 | 1.23 | -3475.578472 |
| 56 | 3.78 | -3475.579531 |
| 60 | 2.36 | -3475.579087 |
| 61 | 1.32 | -3475.578541 |
| 66 | 1.3 | -3475.578527 |
| 75 | 1.12 | -3475.578388 |

**Table S12.** Energies and populations of conformers of diastereomer **l**

| Conformer | Population (%) | E (Hartree) |
|---|---|---|
| 1 | 24.32 | -3475.534006 |
| 2 | 1.6 | -3475.531439 |
| 3 | 9.39 | -3475.533108 |
| 4 | 1.17 | -3475.531142 |
| 8 | 36.79 | -3475.534396 |
| 10 | 13.33 | -3475.533439 |
| 11 | 1.32 | -3475.531258 |
| 17 | 9.72 | -3475.533141 |
| 22 | 1.24 | -3475.531197 |

| | | |
|---|---|---|
| 45 | 1.12 | -3475.531099 |

**Table S13.** Energies and populations of conformers of diastereomer **m**

| Conformer | Population (%) | E (Hartree) |
|---|---|---|
| 1 | 3.49 | -3475.543786 |
| 2 | 2.92 | -3475.543618 |
| 3 | 3.02 | -3475.54365 |
| 4 | 30.07 | -3475.545817 |
| 5 | 1.18 | -3475.542761 |
| 7 | 4.09 | -3475.543934 |
| 9 | 29.5 | -3475.545798 |
| 12 | 1.26 | -3475.542827 |
| 17 | 4.14 | -3475.543946 |
| 18 | 2.96 | -3475.543629 |
| 19 | 3.08 | -3475.543666 |
| 22 | 1.21 | -3475.542786 |
| 31 | 1.33 | -3475.542878 |
| 32 | 3.68 | -3475.543835 |
| 36 | 4.19 | -3475.543957 |
| 37 | 1.26 | -3475.542825 |
| 49 | 1.29 | -3475.542847 |
| 54 | 1.31 | -3475.542862 |

**Table S14.** Energies and populations of conformers of diastereomer **n**

| Conformer | Population (%) | E (Hartree) |
|---|---|---|
| 8 | 3 | -3475.547133 |

| | | |
|---|---|---|
| 9 | 1.09 | -3475.546175 |
| 13 | 4.59 | -3475.547532 |
| 15 | 1.17 | -3475.546244 |
| 16 | 1.29 | -3475.546335 |
| 17 | 3.11 | -3475.547165 |
| 18 | 51.36 | -3475.54981 |
| 20 | 2.01 | -3475.546752 |
| 21 | 5.62 | -3475.547723 |
| 36 | 17.02 | -3475.548769 |
| 38 | 2.14 | -3475.546811 |
| 41 | 1.86 | -3475.546682 |
| 68 | 1.85 | -3475.546674 |
| 72 | 1.86 | -3475.546682 |
| 80 | 2.02 | -3475.54676 |

**Table S15.** Energies and populations of conformers of diastereomer **o**

| Conformer | Population (%) | E (Hartree) |
|---|---|---|
| 1 | 4.78 | -3475.561645 |
| 2 | 4.86 | -3475.56166 |
| 3 | 3.18 | -3475.56126 |
| 4 | 3.13 | -3475.561245 |
| 5 | 3.28 | -3475.56129 |
| 7 | 3.23 | -3475.561274 |
| 8 | 4.77 | -3475.561642 |
| 9 | 7.47 | -3475.562065 |
| 10 | 4.34 | -3475.561553 |
| 11 | 7.33 | -3475.562048 |
| 12 | 2.08 | -3475.56086 |

| | | |
|---|---|---|
| 14 | 2.18 | -3475.560903 |
| 17 | 1.71 | -3475.560676 |
| 20 | 1.91 | -3475.560781 |
| 21 | 1.42 | -3475.560499 |
| 22 | 4.84 | -3475.561655 |
| 23 | 1.24 | -3475.560374 |
| 30 | 2.86 | -3475.56116 |
| 32 | 1.25 | -3475.560378 |
| 34 | 2.55 | -3475.561052 |
| 35 | 2.21 | -3475.560916 |
| 36 | 1.52 | -3475.560563 |
| 38 | 4.46 | -3475.561578 |
| 42 | 1.54 | -3475.560573 |
| 48 | 3.61 | -3475.561379 |
| 49 | 1.66 | -3475.560647 |
| 50 | 1.46 | -3475.560524 |
| 51 | 1.45 | -3475.560518 |
| 53 | 1.91 | -3475.560778 |
| 54 | 1.47 | -3475.560533 |
| 55 | 1.46 | -3475.560522 |
| 58 | 1.46 | -3475.560527 |
| 70 | 2.71 | -3475.561109 |
| 73 | 3.43 | -3475.561331 |
| 75 | 1.25 | -3475.560377 |

**Table S16.** Energies and populations of conformers of diastereomer **p**

| Conformer | Population (%) | E (Hartree) |
|---|---|---|
| 1 | 3.85 | -3475.588999 |

| | | |
|---|---|---|
| 2 | 2.88 | -3475.588726 |
| 3 | 8.2 | -3475.589713 |
| 4 | 1.9 | -3475.588331 |
| 5 | 1.45 | -3475.588082 |
| 6 | 3.84 | -3475.588999 |
| 7 | 4.44 | -3475.589134 |
| 8 | 4.86 | -3475.58922 |
| 9 | 2.88 | -3475.588726 |
| 10 | 3.25 | -3475.588841 |
| 13 | 8.14 | -3475.589706 |
| 16 | 4.2 | -3475.589083 |
| 17 | 1.46 | -3475.588087 |
| 18 | 1.57 | -3475.588157 |
| 20 | 4.88 | -3475.589224 |
| 25 | 4.39 | -3475.589124 |
| 26 | 3.03 | -3475.588775 |
| 28 | 3.27 | -3475.588846 |
| 31 | 2.3 | -3475.588515 |
| 35 | 8.41 | -3475.589737 |
| 36 | 2.12 | -3475.588439 |
| 40 | 2.32 | -3475.58852 |
| 42 | 1.69 | -3475.588224 |
| 45 | 1.36 | -3475.588018 |
| 49 | 1.59 | -3475.588167 |
| 54 | 4.61 | -3475.58917 |
| 56 | 1.02 | -3475.587748 |
| 61 | 3.03 | -3475.588773 |
| 83 | 1.36 | -3475.588016 |
| 92 | 1.69 | -3475.588221 |

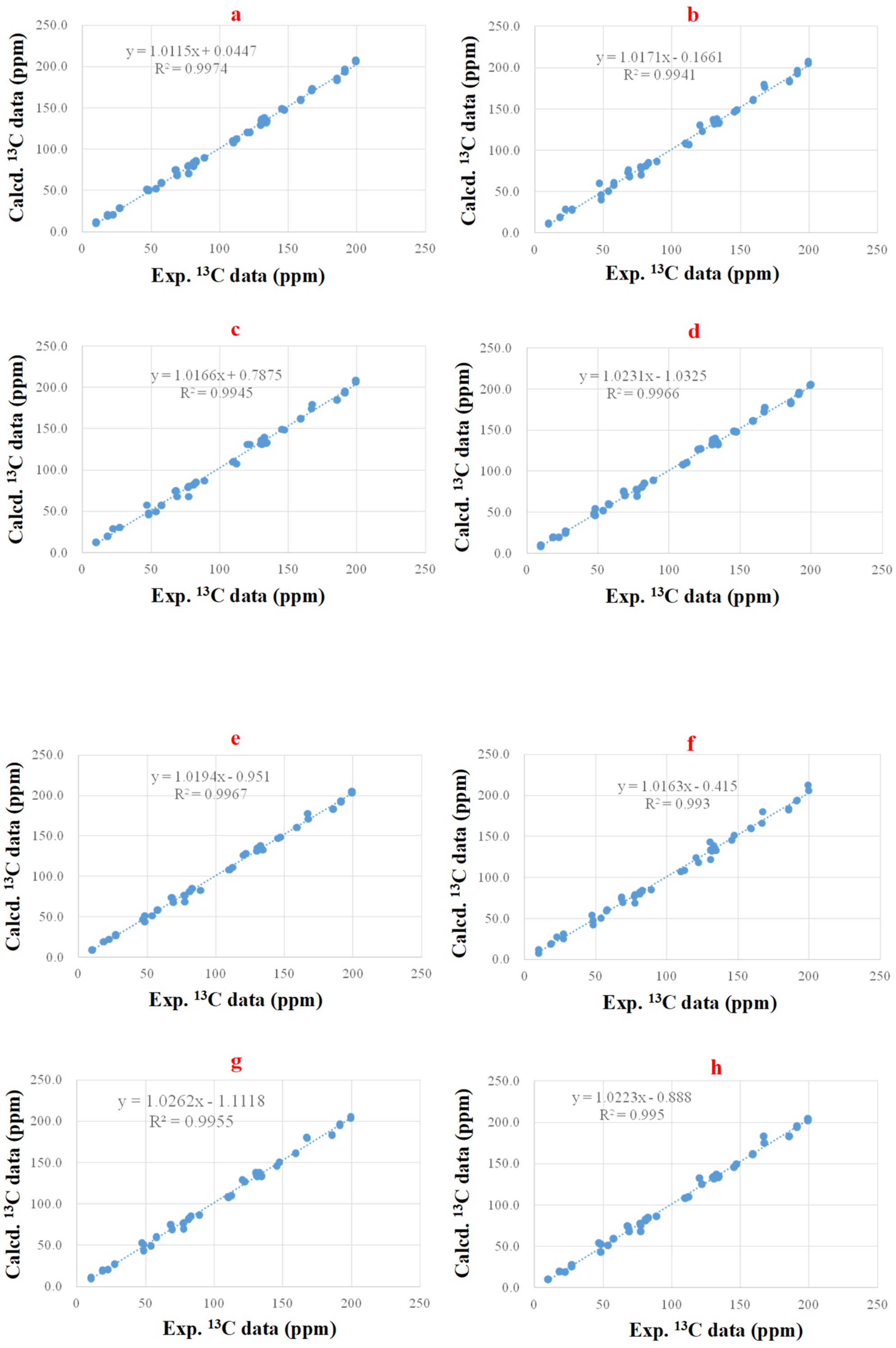

a
y = 1.0115x + 0.0447
R² = 0.9974
b
y = 1.0171x - 0.1661
R² = 0.9941
c
y = 1.0166x + 0.7875
R² = 0.9945
d
y = 1.0231x - 1.0325
R² = 0.9966
e
y = 1.0194x - 0.951
R² = 0.9967
f
y = 1.0163x - 0.415
R² = 0.993
g
y = 1.0262x - 1.1118
R² = 0.9955
h
y = 1.0223x - 0.888
R² = 0.995
Calcd. 13C data (ppm)
Exp. 13C data (ppm)

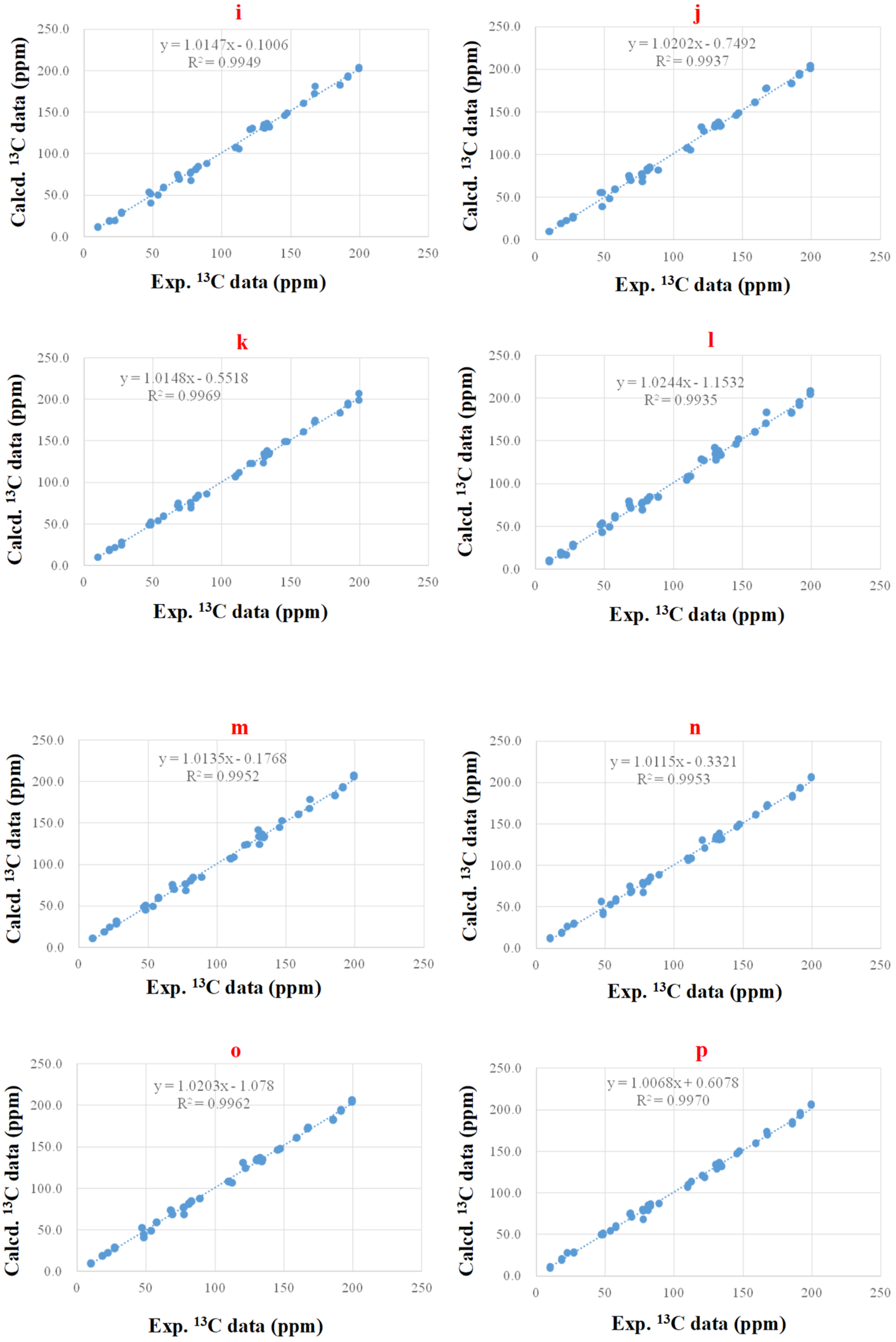


**Figure S10.** Linear regression fitting of computed $^{13}$C NMR chemical shifts of 16 diastereomers of di-isatropolone C (**1**) with experimental data

**Table S17.** The results of experimental and computed $^{13}$C NMR chemical shifts comparison and DP4+ probability analysis of di-isatropolone C (**1**)

| Diastereomer | $R^2$ | RMSE | DP4+ (%) |
|---|---|---|---|
| **a** | 0.9974 | 3.16 | 0.14 |
| **b** | 0.9941 | 4.65 | 0 |
| **c** | 0.9945 | 4.90 | 0 |
| **d** | 0.9966 | 3.75 | 0 |
| **e** | 0.9967 | 3.54 | 0 |
| **f** | 0.9930 | 4.93 | 0 |
| **g** | 0.9955 | 4.33 | 0 |
| **h** | 0.9950 | 4.37 | 0 |
| **i** | 0.9949 | 4.31 | 0 |
| **j** | 0.9937 | 4.76 | 0 |
| **k** | 0.9969 | 3.33 | 0 |
| **l** | 0.9935 | 4.92 | 0 |
| **m** | 0.9952 | 4.09 | 0 |
| **n** | 0.9953 | 3.96 | 0 |
| **o** | 0.9962 | 3.75 | 0 |
| **p** | 0.9970 | 3.33 | 99.86 |

**Table S18.** Conformers of diastereomer **p**

| Conformer | |
|---|---|
| 1 | 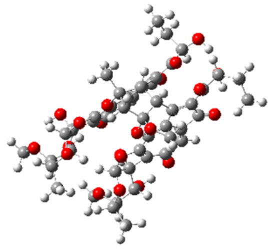 |
| 2 | 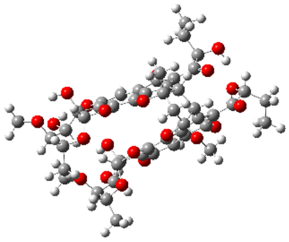 |
| 3 | 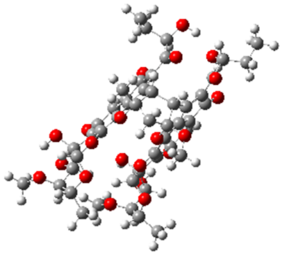 |
| 4 | 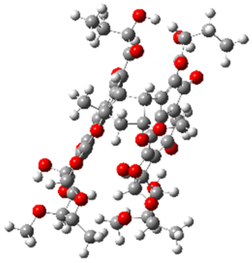 |
| 5 | 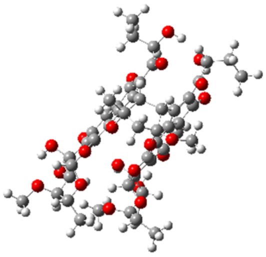 |

| | |
|---|---|
| 6 | 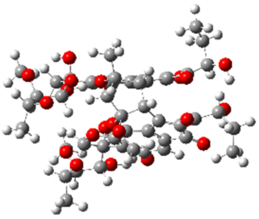 |
| 7 | 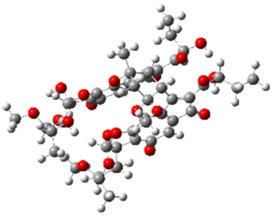 |
| 8 | 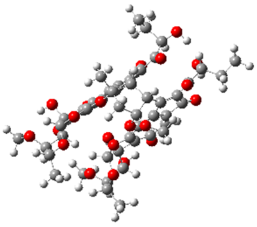 |
| 9 | 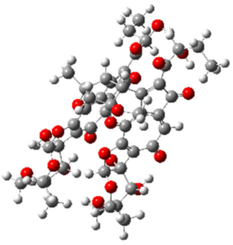 |
| 10 | 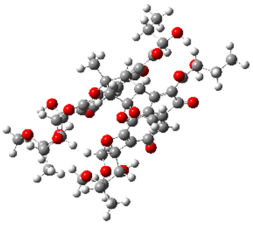 |
| 13 | 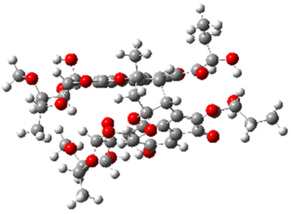 |

| | |
|---|---|
| 16 | 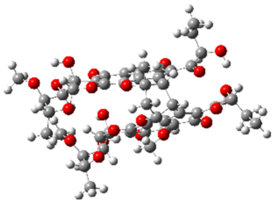 |
| 17 | 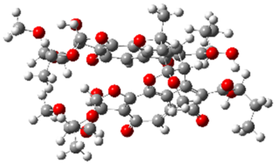 |
| 18 | 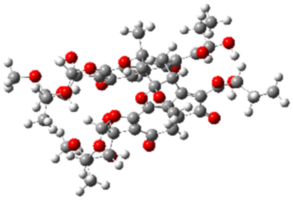 |
| 20 | 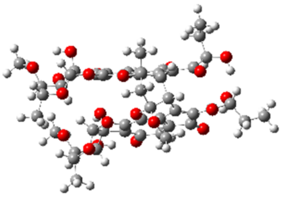 |
| 25 | 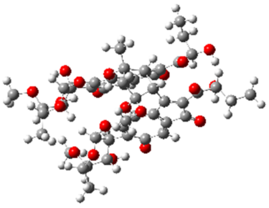 |
| 26 | 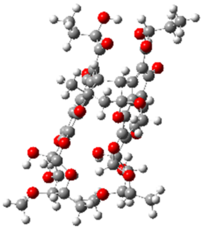 |

| | |
|---|---|
| 28 | 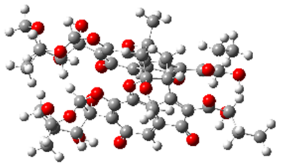 |
| 31 | 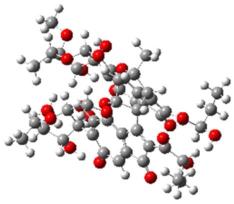 |
| 35 | 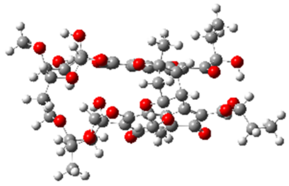 |
| 36 | 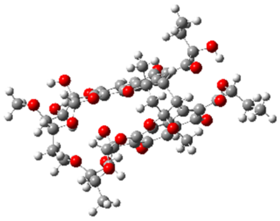 |
| 40 | 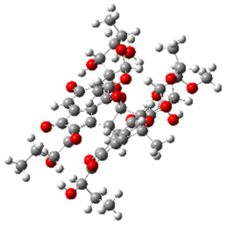 |
| 42 | 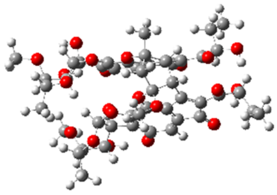 |
| 45 | 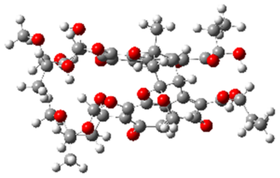 |

| | |
|---|---|
| 49 | 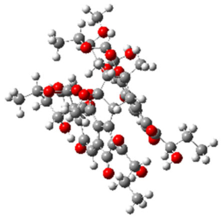 |
| 54 | 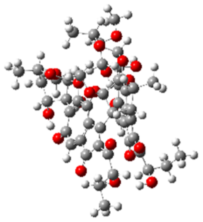 |
| 56 | 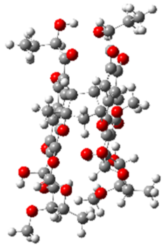 |
| 61 | 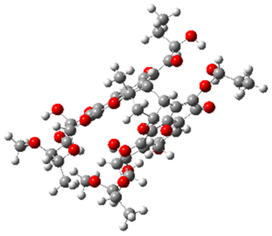 |
| 83 | 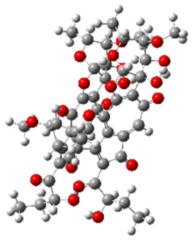 |
| 92 | 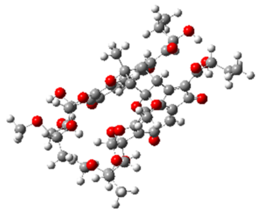 |

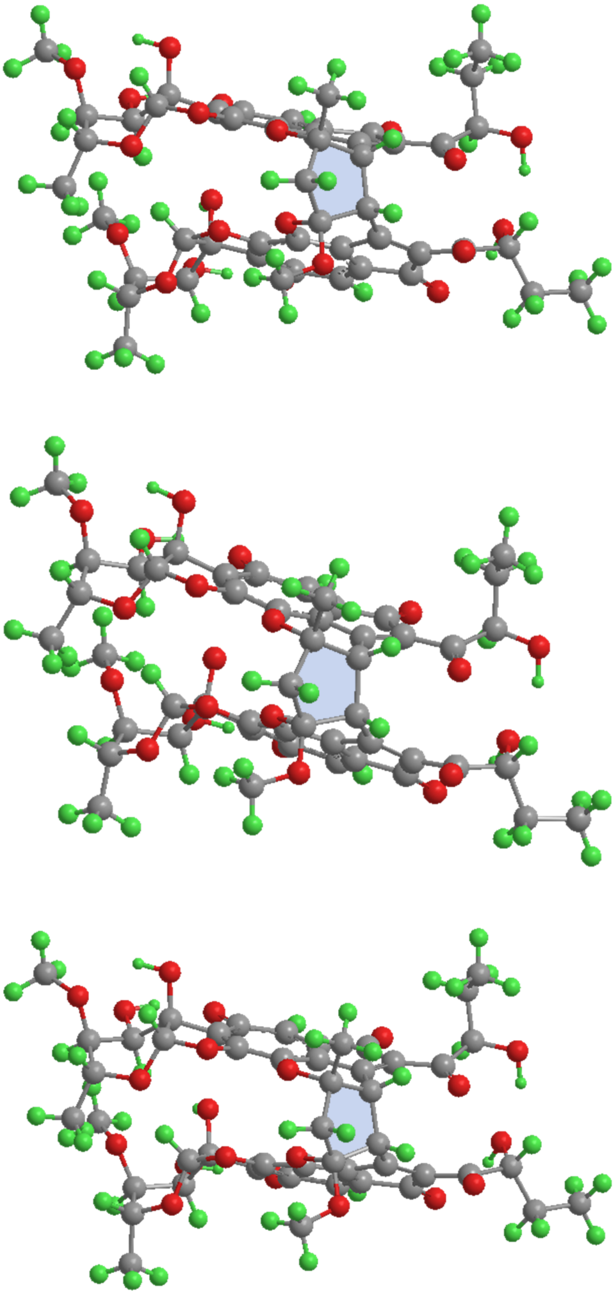

**Figure S11.** The three most populated conformers of diastereomer **p** (from Table S17 and S18)

The cyclopentane ring is blue-lighted to show better that the hydrogen atom attached to C15, methoxy group attached to C16, hydrogen atom attached to C-15" and methyl group attached to C-16" are all out-positioned in the conformers of **p** diastereomer, which may favor close-stacking of the two cyclopentadienone-tropolone planar moieties in di-isatropolone C.

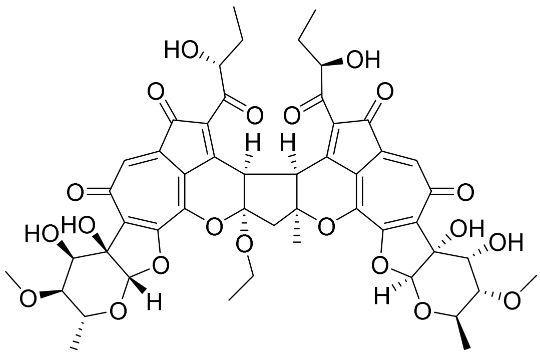


**Figure S12.** The structure of 16-ethoxy di-isatropolone C

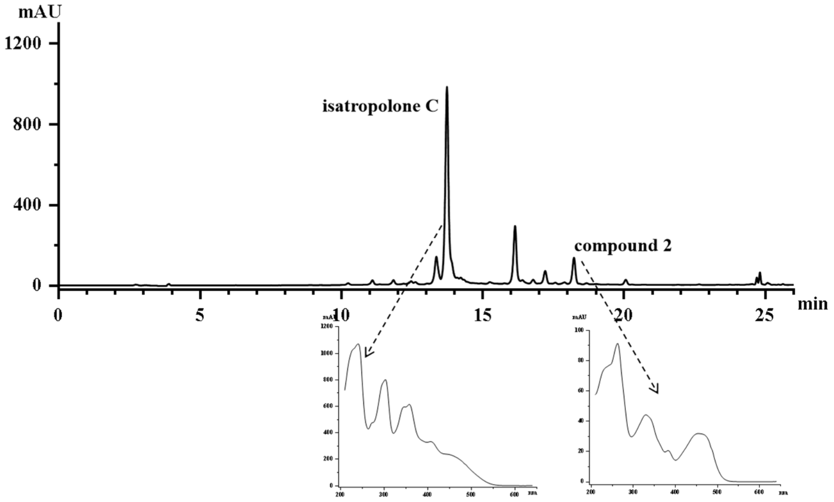


**Figure S13.** HPLC of isatropolone C and 16-ethoxy di-isatropolone C with their UV-visible spectra

Analytical HPLC of isatropolone C after incubation in ethanol for ten days or more revealed a new peak (compound **2**) at 18.2 min (13.8 min for isatropolone C).

## Production and purification of 16-ethoxy di-isatropolone C

Isatropolone C was dissolved in ethanol and incubated at room temperature (20-25 °C) for 20 days. Analytical HPLC of the isatropolone C solution revealed a new peak at 18.2 min. Compound in the new peak was purified by semi-preparative HPLC. A pure preparation of 2.5 mg compound (16-ethoxy di-isatropolone C) was

obtained from 100 mg isatropolone C dissolved in 8.0 ml ethanol. The preparation was used for NMR assays.

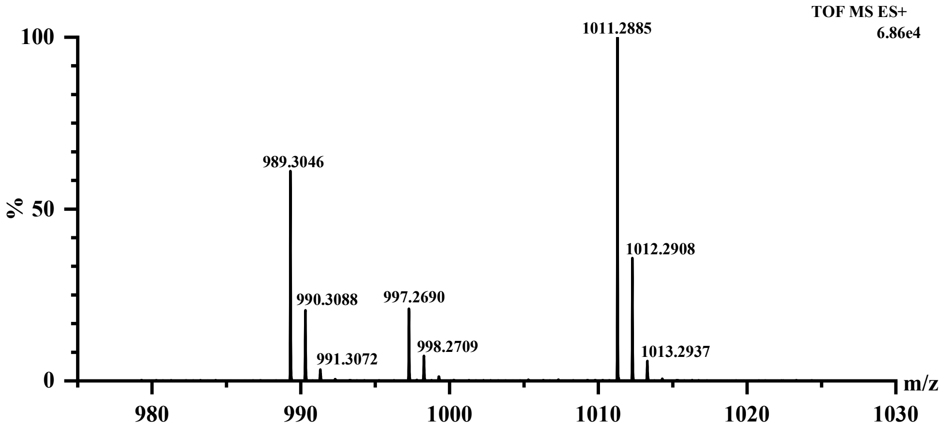


**Figure S14.** HRESIMS of 16-ethoxy di-isatropolone C

**The molecular formula of di-isatropolone C was determined as $C_{50}H_{52}O_{21}$ (*m/z* 989.3046 [M+H]$^+$, calcd for 989.3074)**

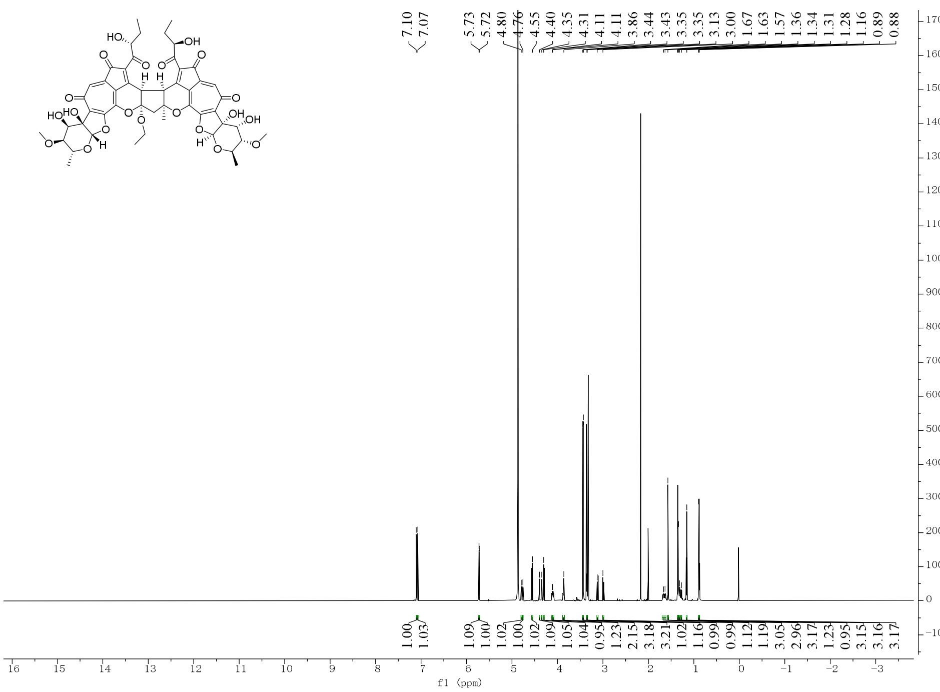

**Figure S15.** $^{1}$H NMR spectrum of 16-ethoxy di-isatropolone C

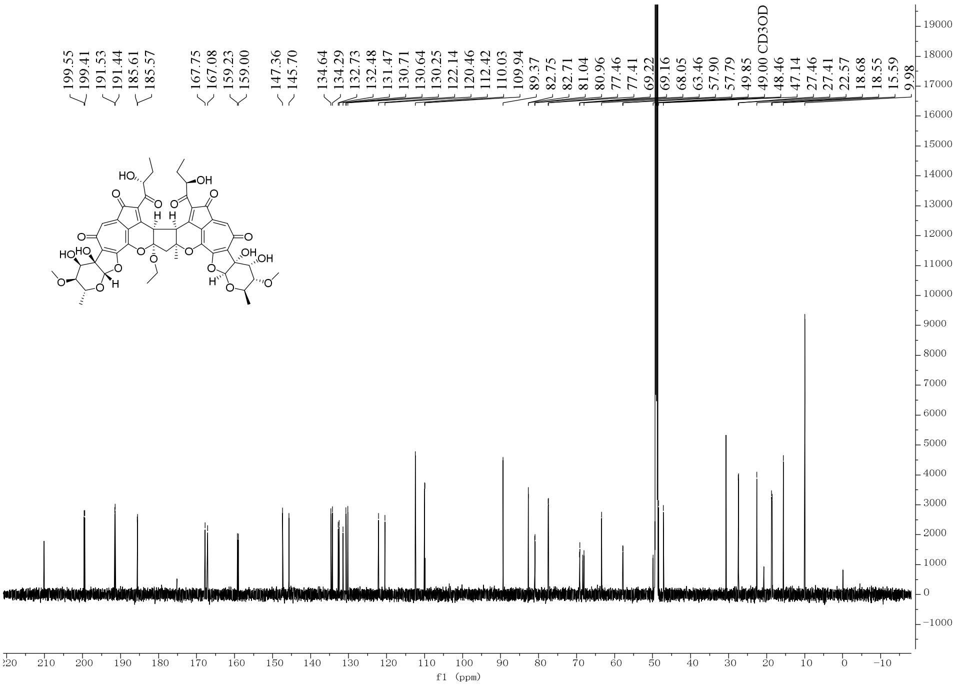

**Figure S16.** $^{13}$C NMR spectrum of 16-ethoxy di-isatropolone C

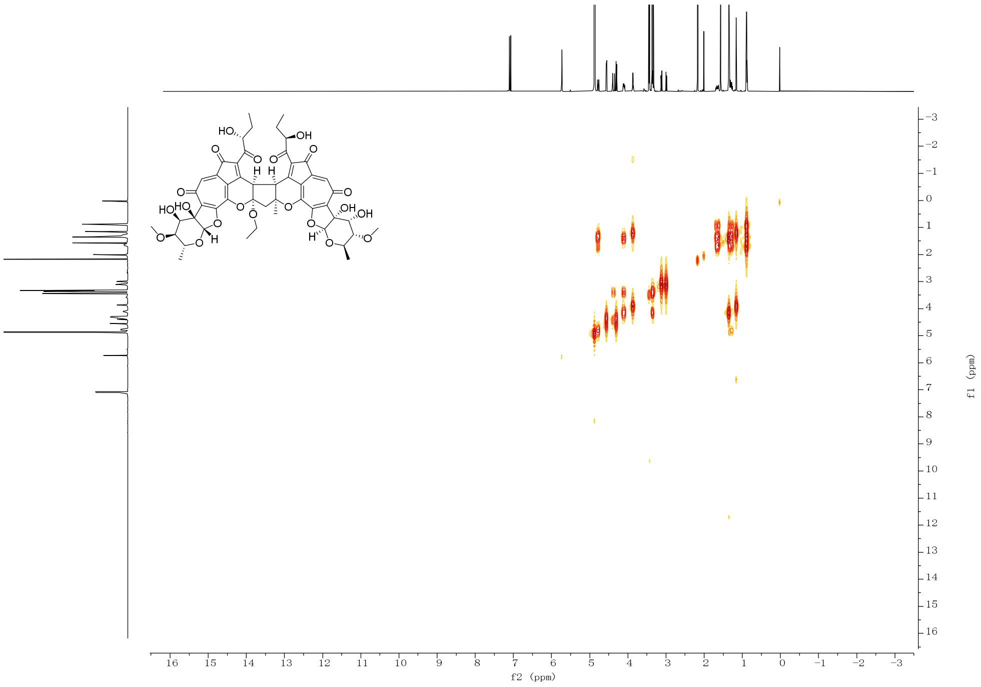

**Figure S17.** $^1H$-$^1H$ COSY spectrum of 16-ethoxy di-isatropolone C

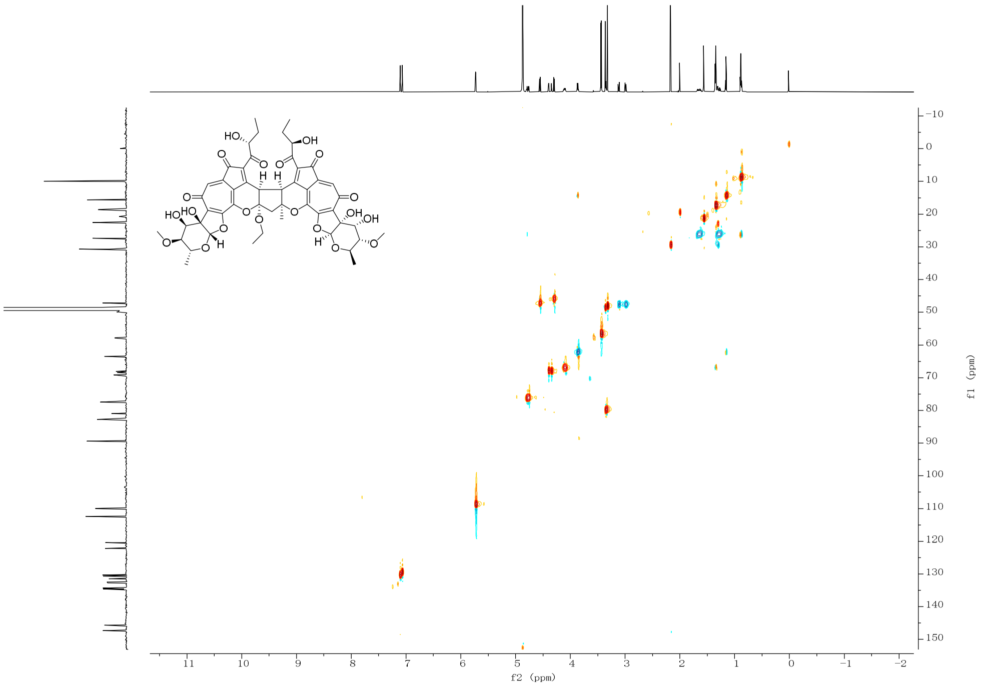

**Figure S18.** HSQC spectrum of 16-ethoxy di-isatropolone C

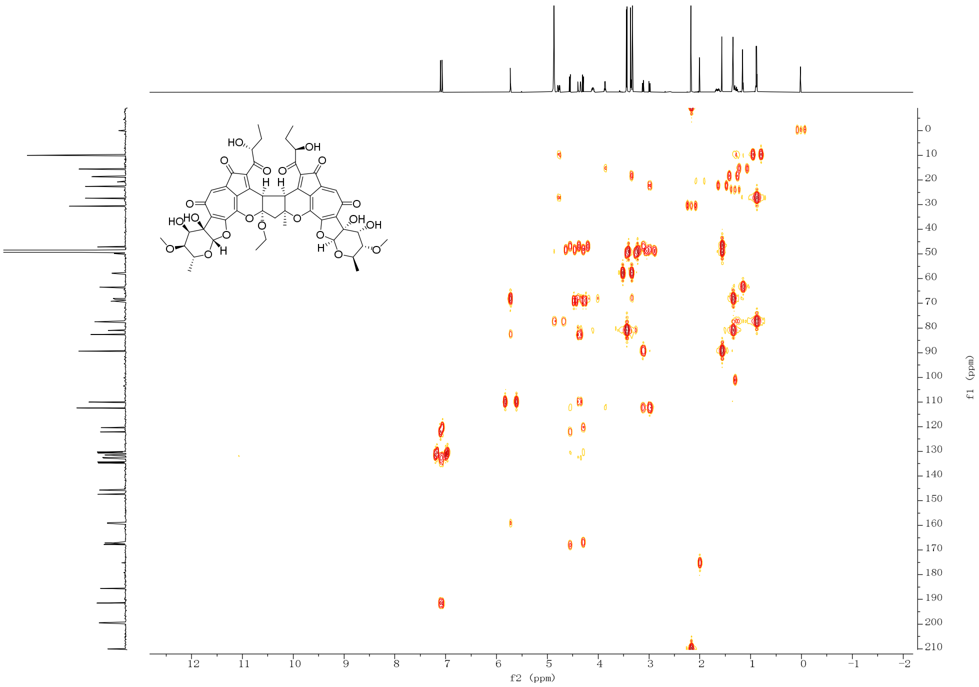

**Figure S19.** HMBC spectrum of 16-ethoxy di-isatropolone C

**Table S19.** NMR data of 16-ethoxy di-isatropolone C in methanol-$d_4$

| Position | $\delta_C$, type | $\delta_H$, mult, ($J$ in Hz) | Position | $\delta_C$, type | $\delta_H$, mult, ($J$ in Hz) |
|---|---|---|---|---|---|
| 1 | 10.0, $CH_3$ | 0.88 t (7.2) | 1'' | 10.0, $CH_3$ | 0.89 t (7.2) |
| 2 | 27.4, $CH_2$ | 1.27 m, 1.65 m | 2'' | 27.5, $CH_2$ | 1.31 m, 1.67 m |
| 3 | 77.4, CH | 4.76 dd (8.0, 4.0) | 3'' | 77.5, CH | 4.79 dd (8.0, 4.0) |
| 4 | 199.4, C | | 4'' | 199.6, C | |
| 5 | 130.2, C | | 5'' | 130.7, C | |
| 6 | 191.4, C | | 6'' | 191.5, C | |
| 7 | 130.6, CH | 7.07 s | 7'' | 131.5, CH | 7.15 s |
| 8 | 185.6, C | | 8'' | 185.6, C | |
| 9 | 132.5, C | | 9'' | 132.7, C | |
| 10 | 159.0, C | | 10'' | 159.2, C | |
| 11 | 145.7, C | | 11'' | 147.4, C | |

| | | | | | |
|---|---|---|---|---|---|
| 12 | 134.3, C | | 12" | 134.6, C | |
| 13 | 120.5, C | | 13" | 122.1,C | |
| 14 | 167.1, C | | 14" | 167.8, C | |
| 15 | 47.1, CH | 4.30 d (12.8) | 15" | 48.5, CH | 4.55 d (12.8) |
| 16 | 112.4, C | | 16" | 89.4, C | |
| 17 | 48.8, $CH_2$ | 2.99 d (16.0)<br>3.12 d (16.0) | 17" | 22.6, $CH_3$ | 1.57 s |
| 18 | 63.4, $CH_2$ | 3.87 m | | | |
| 19 | 15.6, $CH_3$ | 1.16 t (7.2) | | | |
| 1' | 109.9, CH | 5.72 d (5.6) | 1''' | 110.0, CH | 5.72 d (5.6) |
| 2' | 82.7, C | | 2''' | 82.8, C | |
| 3' | 69.2, CH | 4.35 d (3.2) | 3''' | 69.2, CH | 4.40 d (2.4) |
| 4' | 81.0, CH | 3.35 m overlap | 4''' | 81.0, CH | 3.37 m overlap |
| 5' | 68.0, CH | 4.10 m | 5''' | 68.4, CH | 4.12 m |
| 6' | 18.6, $CH_3$ | 1.35 d (6.4) | 6''' | 18.7, $CH_3$ | 1.36 d (6.4) |
| 7' | 57.9, $CH_3$ | 3.43 s | 7''' | 58.0, $CH_3$ | 3.45 s |

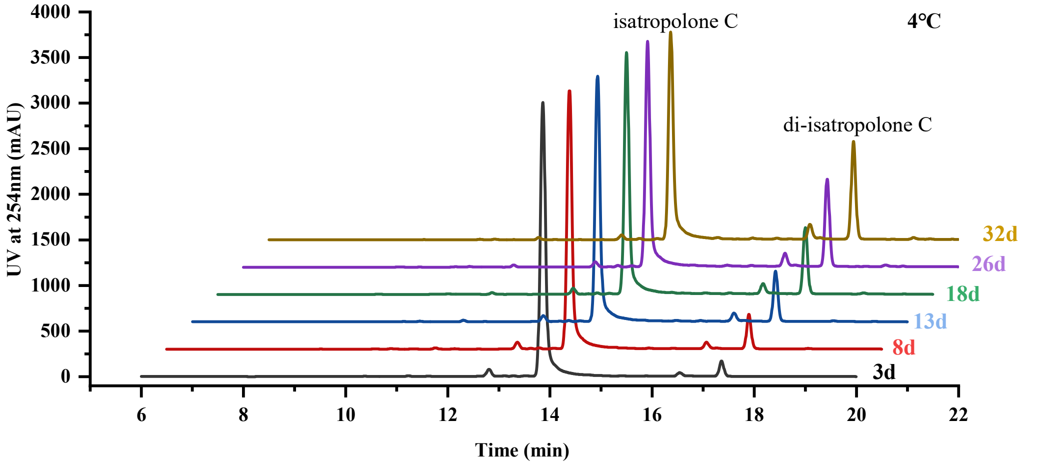


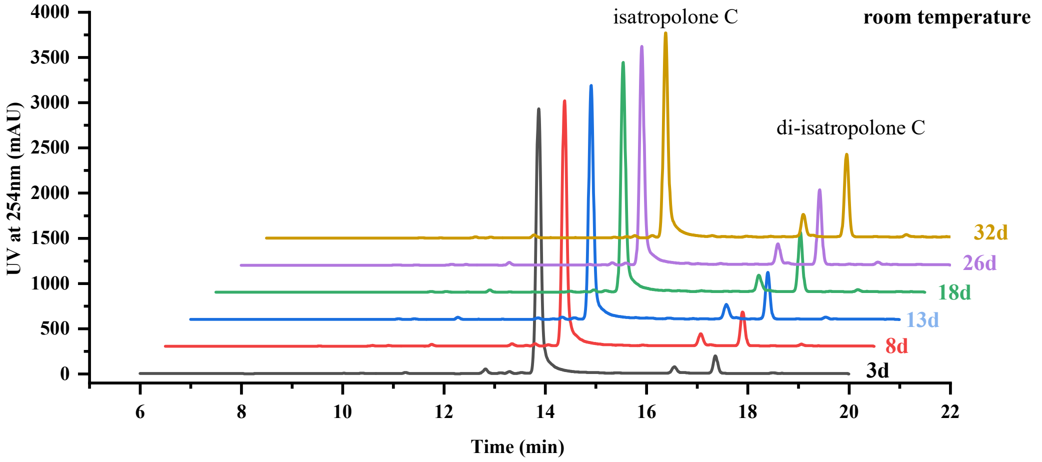


**Figure S20.** A time-course monitoring of di-isatropolone C production from isatropolone C in methanol incubated at 4 °C or room temperature

Isatropolone C was dissolved in 2.0 mL methanol at a concentration of 2.0 mg/mL, then divided into two equal parts. One part was incubated at 4 °C for a period of over one month, while the other part was incubated at room temperature (20-25 °C) for the same time. Each part was periodically sampled for di-isatropolone C production by analytical HPLC (each with an injection volume of 10 μL).

**Di-isatropolone C productions increased with incubation time but displayed no significant differences at 4 °C and room temperature.**

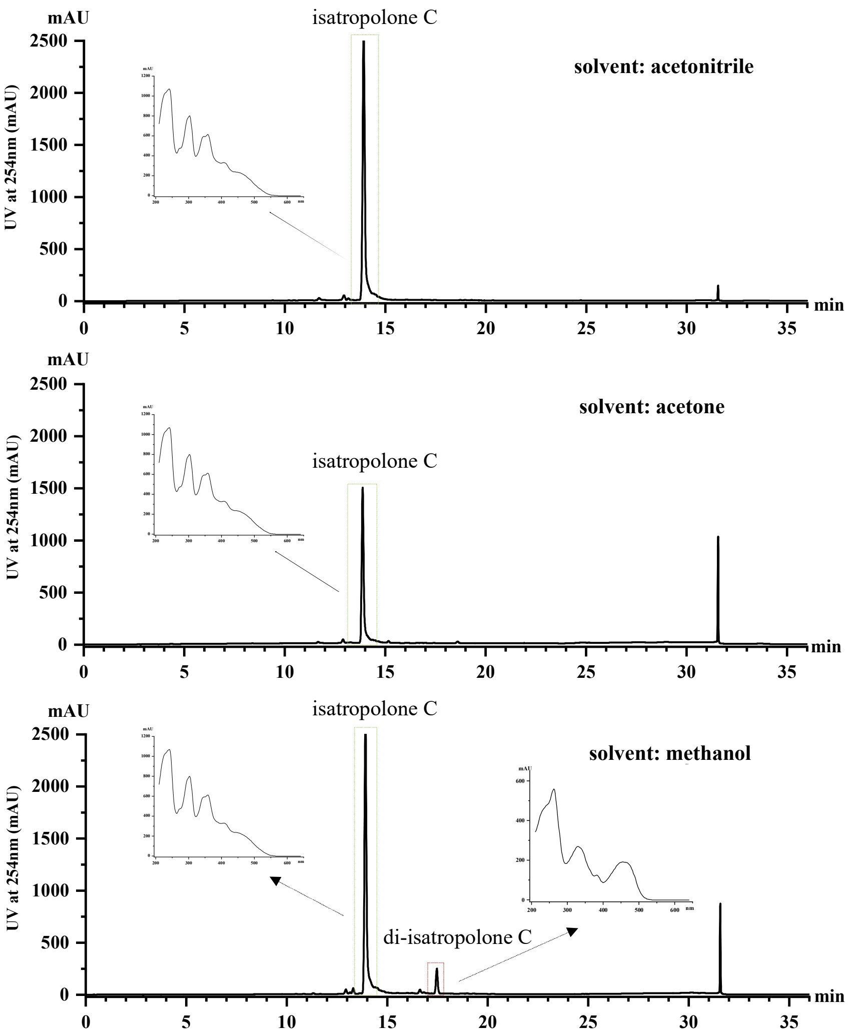


**Figure S21.** Production of di-isatropolone C from isatropolone C in methanol, acetonitrile, or acetone

Isatropolone C was dissolved in 400 μL methanol, acetonitrile or acetone at a concentration of 0.8 0.8 and 0.4 mg/mL, respectively. The solutions were incubated at 4 °C for 30 days and then analyzed by HPLC for di-isatropolone C production (each with an injection volume of 30 μL).

**Di-isatropolone C was not observed in isatropolone C dissolved in acetonitrile or acetone.**

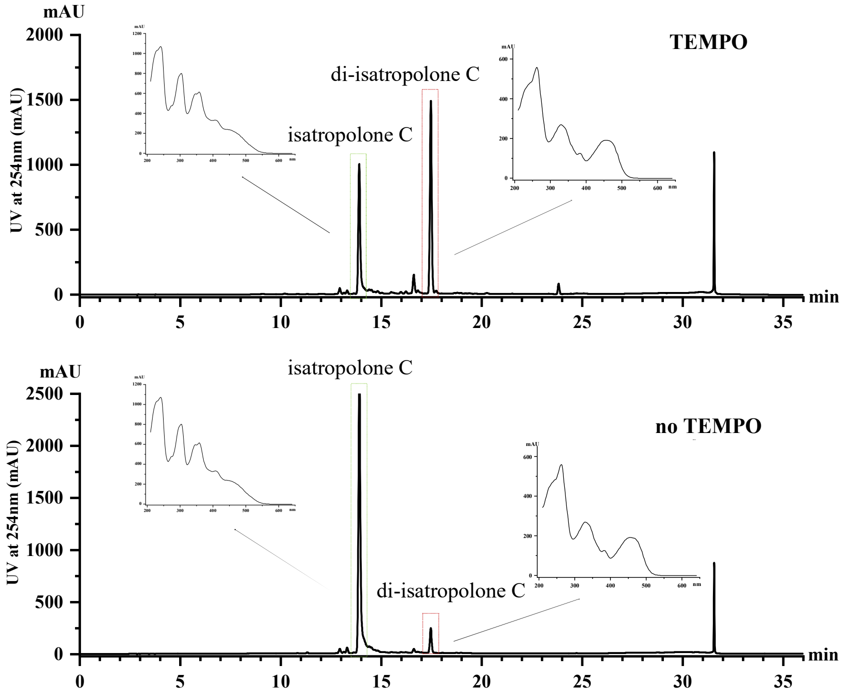


**Figure S22.** Production of di-isatropolone C from isatropolone C in methanol with TEMPO

Isatropolone C was dissolved in methanol at a concentration of 0.8 mg/mL, and TEMPO was dissolved in $H_2O$ at a concentration of 10.0 mg/mL. The isatropolone C solution (200 μL) was added with 1.0 μL TEMPO solution. The mixed solution was placed at 4 °C for 30 days and then analyzed by HPLC for di-isatropolone C production (with an injection volume of 20 μL). Meanwhile, an identical volume of isatropolone C solution without TEMPO was also placed at 4 °C for 30 days and then analyzed by HPLC for di-isatropolone C production as control.

**TEMPO increased significantly (about six folds) the production of di-isatropolone C**.

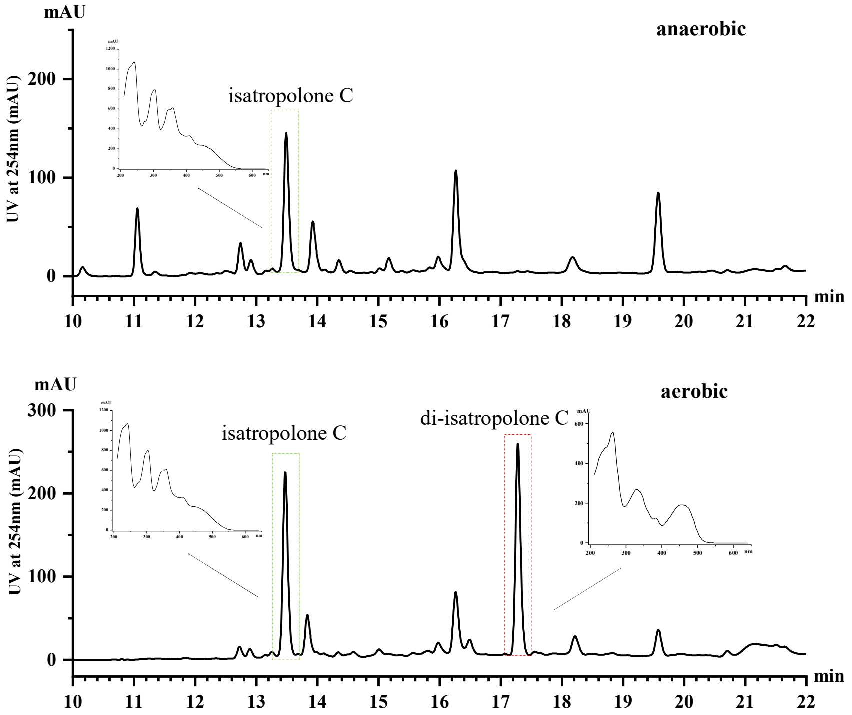


**Figure S23.** Production of di-isatropolone C from isatropolone C in methanol under air (aerobic) or oxygen-free gas mixture (anaerobic)

Isatropolone C was dissolved in degassing methanol at a concentration of 0.8 mg/mL, and TEMPO was dissolved in degassing $H_2O$ at a concentration of 10.0 mg/mL. The isatropolone C solution (400 μL) was added with 5.0 μL TEMPO solution. The mixed solution was incubated anaerobically (under a gas mixture of nitrogen 90%, carbon dioxide 5% and hydrogen 5%) at 37 °C for 30 days and then analyzed by HPLC for di-isatropolone C production (with an injection volume of 1.0 μL). Meanwhile, an identical volume of the mixed solution was incubated aerobically (under air) at 37 °C for 30 days as control.

**Di-isatropolone C was not observed in isatropolone C solution incubated anaerobically.**
